# Effect of Sortilin1 on promoting angiogenesis and systemic metastasis in hepatocellular carcinoma via the Notch signaling pathway and CD133

**DOI:** 10.1038/s41419-024-07016-7

**Published:** 2024-08-29

**Authors:** Hye Ri Ahn, Sujin Kim, Geum Ok Baek, Moon Gyeong Yoon, Minji Kang, Jestlin Tianthing Ng, Yunjin Go, Su Bin Lim, Jung Hwan Yoon, Jee-Yeong Jeong, Ji Eun Han, Soon Sun Kim, Jae Youn Cheong, Jung Woo Eun, Hyo Jung Cho

**Affiliations:** 1 Department of Gastroenterology, Ajou University School of Medicine, Suwon, South Korea; 2 Department of Biomedical Sciences, Ajou University Graduate School of Medicine, Suwon, South Korea; 3 Department of Biochemistry & Molecular Biology, Ajou University School of Medicine, Suwon, South Korea; 4 Department of Pathology College of Medicine, The Catholic University of Korea, Seoul, South Korea; 5 Department of Biochemistry College of Medicine, Kosin University Gamchen-ro, Busan, South Korea

**Keywords:** Oncogenes, Epithelial-mesenchymal transition, DNA methylation

## Abstract

Hepatocellular carcinoma (HCC) is known to be lethal disease. However, its prognosis remains poor, primarily because the precise oncogenic mechanisms underlying HCC progression remain elusive, thus hampering effective treatment. Here, we aimed to identify the potential oncogenes in HCC and elucidate the underlying mechanisms of their action. To identify potential candidate genes, an integrative analysis of eight publicly available genomic datasets was performed, and the functional implications of the identified genes were assessed in vitro and in vivo. Sortilin 1 (*SORT1*) was identified as a potential candidate oncogene in HCC, and its overexpression in HCC cells was confirmed by analyzing spatial transcriptomic and single-cell data. Silencing *SORT1* in Huh-7 and Hep3B cells significantly reduced HCC progression in vitro and in vivo. Functional analyses of oncogenic pathways revealed that *SORT1* expression regulated the Notch signaling pathway activation and CD133 expression. Furthermore, analysis of epigenetic regulation of the candidate gene and its clinical implications using The Cancer Genome Atlas Liver Hepatocellular Carcinoma (TCGA LIHC) and our HCC cohort (AJOU_HCC cohort) data demonstrated an inverse correlation between the methylation status of the *SORT1* promoter region, specifically at the cg16988986 site, and *SORT1* mRNA expression, indicating the epigenetic regulation of *SORT1* in HCC. In addition, the distinct methylation status of cg16988986 was significantly associated with patient survival. In conclusion, *SORT1* plays a pivotal role in HCC by activating the Notch signaling pathway and increasing CD133 expression. These findings suggest *SORT1* as a promising therapeutic target for HCC.

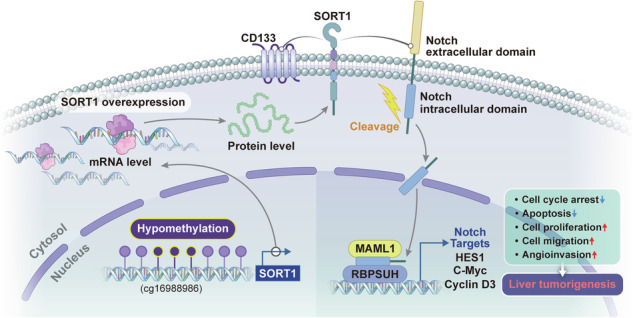

## Introduction

Hepatocellular carcinoma (HCC) is a highly lethal malignancy with poor prognosis, primarily because of its propensity for metastasis and recurrence [1]. Despite recent advances in immune checkpoint inhibitors and targeted therapies, the survival of patients with advanced-stage HCC remains disappointingly short [2]. Moreover, the fundamental mechanisms underlying HCC development and progression have not been fully elucidated. Therefore, further investigation is imperative to gain in-depth insights into the mechanisms underlying HCC progression and develop more effective therapeutic strategies that hold promise for improved outcomes and increased survival of patients with advanced-stage HCC [3]. Accordingly, research aimed at identifying the key genes and molecular pathways that drive hepatocarcinogenesis is crucial.

Sortilin 1 (SORT1), encoded by a gene located on chromosome 1p13.3, is a crucial scavenging receptor that belongs to the vacuolar protein-sorting 10 protein (Vps10p) family. SORT1 comprises an extracellular Vps10p domain, a single transmembrane domain, and a short cytoplasmic tail [4, 5]. SORT1 plays a dual role in endocytosis and receptor trafficking in the plasma membrane and subcellular compartments, such as the trans-Golgi network, endosomes, and lysosomes [5, 6]. Furthermore, *SORT1* is overexpressed in cancer tissues and has been implicated in promoting cancer cell proliferation and survival [7], thus making it a promising therapeutic target for various cancers, including breast and ovarian cancers. However, comprehensive studies on the role and oncogenic mechanisms of action of *SORT1* in HCC are limited.

In this study, we aimed to explore whether *SORT1* is a pivotal oncogene underlying HCC progression and investigate its molecular mechanism of action. The findings of this study could provide insights into the molecular basis of HCC pathogenesis and could help identify therapeutic targets with potential applications in precision medicine.

## Results

### *SORT1* is a potential oncogenic driver gene in HCC progression

**Fig. 1:**
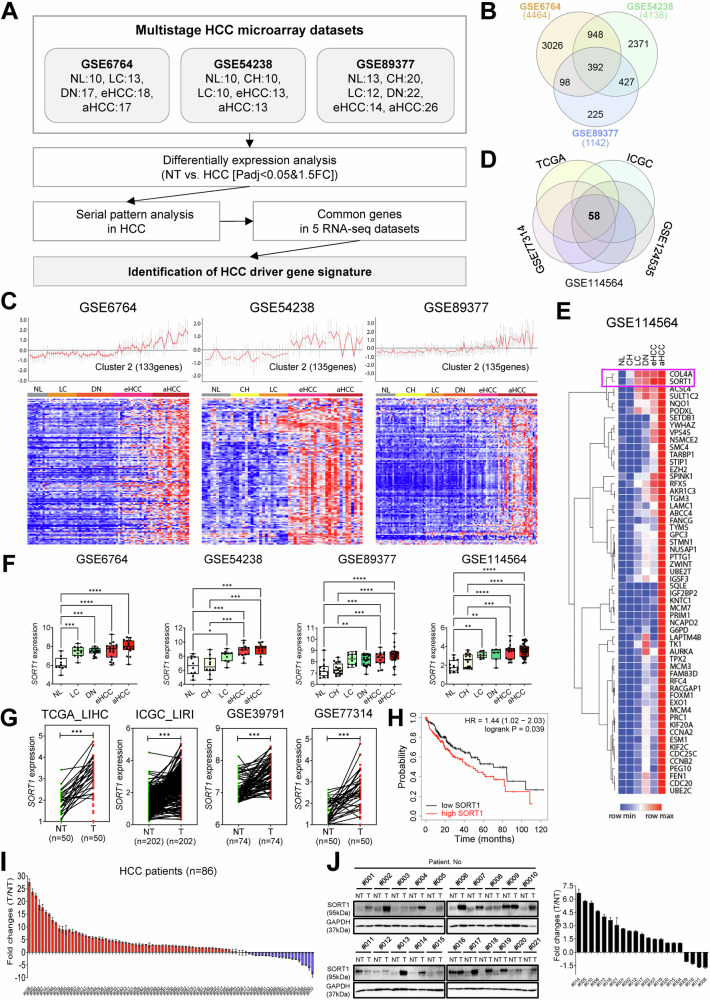
Analysis of hepatocellular carcinoma (HCC) driver gene signatures across multiple datasets. **A** Flow diagram detailing the methodology used to identify HCC driver gene signatures. **B** Venn diagram illustrating the overlap of significantly differentially expressed genes across the three datasets. **C** Serial pattern analysis of each dataset. The upper panels show line plots of gene expression patterns in different sample groups (NL, CH, LC, DN, eHCC, and aHCC) on the X-axis. The bottom panels display heat maps of the gene expression levels. NL, normal liver; CH, chronic hepatitis; LC, liver cirrhosis; DN, dysplastic nodules; eHCC, early HCC; aHCC, advanced HCC. **D** Venn diagram showing the overlap of common genes across the five RNA-seq datasets (TCGA, ICGC, GSE77314, GSE114564, and GSE124535). **E** Heatmap of the GSE114564 dataset showing gene expression patterns. Different genes are listed on the right side of the heatmap. **F** Box plots showing *SORT1* expression at various stages of liver disease and cancer progression across the four datasets. The Y-axis represents *SORT1* expression, whereas the X-axis represents different sample groups. **G** Paired plots showing the expression of *SORT1* in HCC tumor (T) vs. non-tumoral (NT) tissues across four datasets: The Cancer Genome Atlas Liver Hepatocellular Carcinoma (TCGA LIHC), ICGC LIRI, GSE37991, and GSE77314. Each line connects the paired NT and T samples with a significant *SORT1* upregulation in the tumor samples. **H** Kaplan–Meier survival analysis curve displaying survival probability over time for two groups: low and high *SORT1* expression. The high *SORT1* group showcases a reduced survival probability compared to the low *SORT1* group, indicating the potential role of *SORT1* as a prognostic marker. The hazard ratio (HR) of 1.44 (95% confidence interval (CI): 1.02–2.03) with a log-rank *p*-value of 0.039 suggests a significant association between high *SORT1* expression and poor prognosis. **I** Bar graph showing fold-changes in *SORT1* expression across a cohort of patients with HCC (n = 86), highlighting its differential expression in HCC. **J** Western blot analysis and quantitative optical density of SORT1 in paired HCC and adjacent normal tissues obtained from various patients. Glyceraldehyde 3-phosphate dehydrogenase (GAPDH) was used as the loading control. Statistical significance is indicated as *p < 0.05; **p < 0.01; ***p < 0.001; ****p < 0.0001. The analysis was performed using one-way ANOVA.

First, we analyzed the differentially expressed genes (DEGs) in HCC tissues compared to those in non-tumor tissues using publicly available omics datasets to identify the key genes associated with hepatocarcinogenesis (Fig. 1A and Supplementary Fig. 1A). DEG expression distinctly divided the tumor and non-tumor samples into Uniform Manifold Approximation and Projection plots across the three datasets (Supplementary Fig. 1B). Using a Venn diagram analysis, 392 DEGs that exhibited consistent expression patterns across all three datasets were identified (Fig. 1B). Subsequently, serial pattern analysis was performed to identify gene signatures that demonstrated a progressive increase in expression with disease progression from normal liver tissue to various stages, including liver cirrhosis, dysplastic nodules, early HCC, and advanced HCC (Fig. 1C, Supplementary Table 1). The analysis revealed 125 genes that exhibited a consistent increase in expression patterns with disease progression in all three datasets (Supplementary Fig. 1C). Further enrichment analysis of these genes revealed their association with cancer-associated mechanisms (Supplementary Fig. 1D). Subsequently, to refine the selection of key genes involved in hepatocarcinogenesis, we assessed the expression of these 125 genes in five additional publicly available RNA sequencing datasets. The analysis identified 58 genes that consistently exhibited a gradual increase in expression with disease progression across all five datasets (Fig. 1D, Supplementary Table 2). The expression of these 58 genes according to disease stage in GSE114564 is shown in Fig. 1E, among which *COL4A1* and *SORT1* displayed substantial upregulation with HCC progression. Several studies have demonstrated the oncogenic role of *COL4A1* in HCC growth and metastasis [8–10]; however, the role of *SORT1* in HCC has remained unexplored.

Our findings demonstrated a gradual increase in *SORT1* expression as the disease progressed across the four multi-stage datasets (Fig. 1F) and confirmed the *SORT1* overexpression in HCC tissues compared to that in non-tumor tissues (Fig. 1G). Additionally, patients with elevated *SORT1* expression showed unfavorable prognostic outcomes in The Cancer Genome Atlas Liver Hepatocellular Carcinoma (TCGA LIHC) dataset (Fig. 1H).

Furthermore, *SORT1* overexpression in HCC was verified in the AJOU_HCC cohort. As shown in Fig. 1I, *SORT1* was significantly upregulated in 63 of 86 (73.26%) samples from patients with HCC compared with that in their non-tumorous counterparts. Furthermore, western blot analysis revealed that 17 of the 21 pairs (80.95%) exhibited higher *SORT1* expression in HCC tissue samples than in the adjacent non-tumor tissue samples (Fig. 1J). To further validate SORT1 overexpression at the tissue level, we supplemented our bioinformatic analysis and western blot results with immunohistochemistry (IHC) data obtained from the Human Protein Atlas. Supplementary Fig. 1E presents representative IHC images along with a bar chart showing the staining ratio. The bar chart illustrates a significant increase in SORT1 staining in HCC tissues compared to non-tumorous tissues, further corroborating our previous findings and strengthening the evidence for SORT1 as an oncogenic driver in HCC progression.

### *SORT1* is overexpressed in malignant cells

**Fig. 2:**
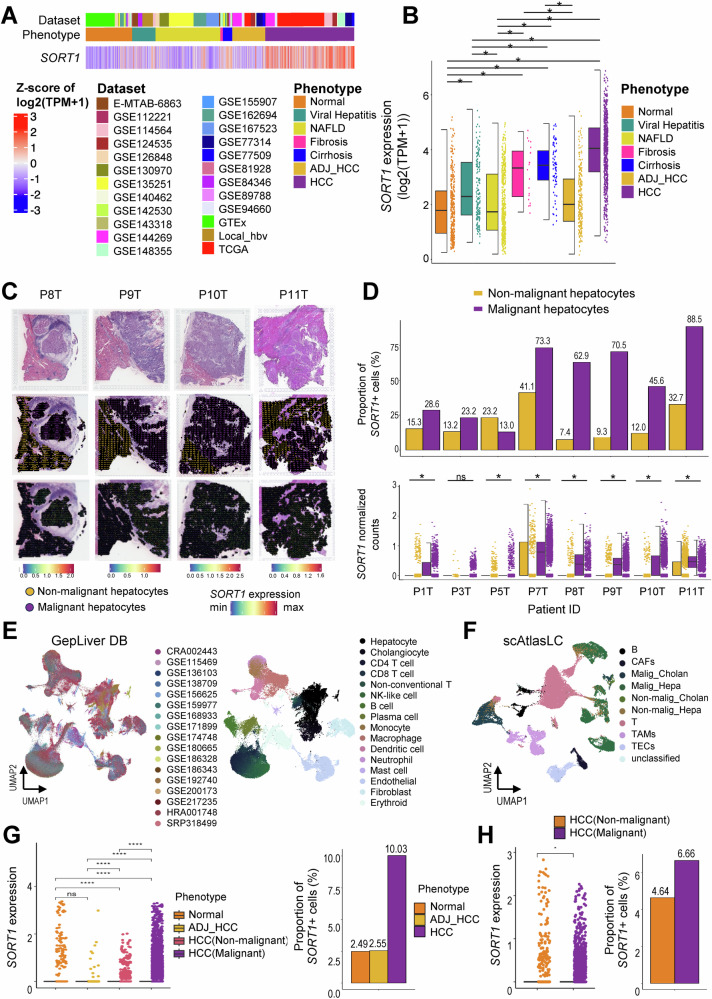
Verification of *SORT1* overexpression in hepatocellular carcinoma (HCC) cells using the GepLiver and the Mendeley Databases (DBs). **A** Heatmap of *SORT1* expression (z-score) in 24 datasets from the GepLiver DB. **B** *SORT1* expression (log_2_(TPM + 1)) across tissues with different phenotypes. **C** Top: Representative hematoxylin and eosin (H&E) images. Middle: Spatial transcriptomics (ST) spots indicated by non-malignant (yellow) and malignant (purple) hepatocytes from tumor tissues. Bottom: *SORT1* expression in the spatial sections. P: patient, T: tumor tissue. **D** Proportion of *SORT1*^+^ cells (%) (top) and *SORT1* expression (bottom) in all analyzed tumor tissues. P: patient, T: tumor tissue. **E** UMAP plot of the GepLiver DB, an integrated liver scRNA-seq dataset. Each cell is colored according to the dataset (left) and major cell type (right). **F** UMAP plot of scAtlasLC. Each cell is colored by major cell type. **G** *SORT1* expression (left) and proportion of *SORT1*^+^ cells (%) in hepatocytes of the GepLiver DB across different phenotypes. **H** *SORT1* expression (left) and proportion of *SORT1*^+^ cells (%) in hepatocytes of the Single-cell Atlas in Liver Cancer (scAtlasLC) across different phenotypes. Statistical significance is indicated as ns, non-significant; *p < 0.05; ****p < 0.0001. Analyses were performed using the Welch’s *t*-test and one-way ANOVA.

*SORT1* overexpression was consistently observed in HCC across eight publicly available omics datasets and in the AJOU_HCC cohort. To validate *SORT1* overexpression in malignant cells at the single-cell level, we conducted an additional analysis using GepLiver DB, an integrative liver expression atlas covering developmental and liver disease phases [11]. Our findings confirmed *SORT1* overexpression in HCC, using bulk RNA-seq data (Fig. 2A). Multi-stage analysis revealed the lowest *SORT1* expression in normal liver tissues, which gradually increased as the liver disease progressed from viral hepatitis to hepatic fibrosis, cirrhosis, and HCC (Fig. 2B). Additionally, 10X Visium-derived spatial transcriptomic data obtained from Mendeley demonstrated significant *SORT1* overexpression in malignant hepatocytes compared to that in non-malignant hepatocytes (Fig. 2C, D) [12]. Single-cell analysis of the GepLiver DB, comprising 17 single-cell RNA-seq (scRNA-seq) datasets, confirmed *SORT1* overexpression in malignant HCC cells. The *SORT1*^+^ cell proportion was notably higher in HCC cells than in normal and adjacent non-tumor cells (Fig. 2E, G). This result was consistently verified using the Single-Cell Atlas for Liver Cancer (scAtlasLC; Fig. 2F, H) [13].

### Functional implications of *SORT1* in HCC: impact on proliferation, colony formation, migration, apoptosis, and cell cycle dynamics

**Fig. 3:**
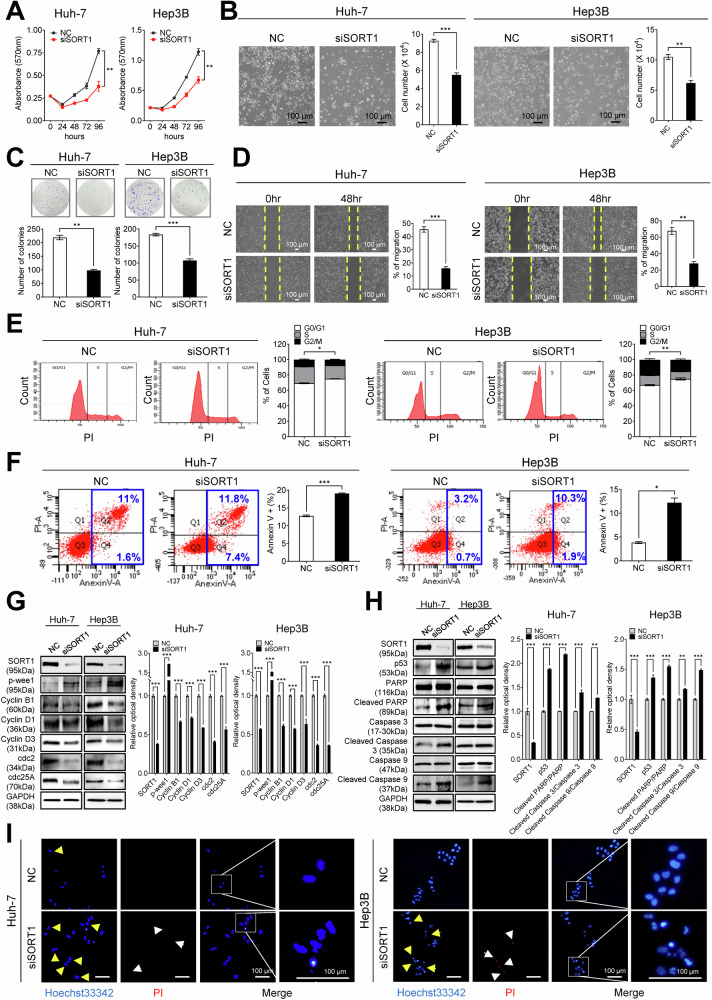
Functional implications of *SORT1* in hepatocellular carcinoma (HCC): impact on proliferation, colony formation, migration, apoptosis, and cell cycle dynamics. **A** Proliferation curves of Huh-7 and Hep3B cells post-treatment with NC and siSORT1 monitored over 96 h. **B** Microscopic images showing the morphology of Huh-7 and Hep3B liver cancer cell lines after treatment with negative control (NC) or siSORT1. The bar graphs on the right quantify the cell numbers for each condition. **C** Colony formation assay for Huh-7 and Hep3B cells treated with NC or siSORT1. The number of colonies is quantified in the bar graphs. **D** Wound-healing assay showing the migratory potential of Huh-7 and Hep3B cells in response to *SORT1* silencing at 0 and 48 h post-wound creation. Quantification of wound closure is shown in bar graphs on the right. **E** Cell cycle distribution of Huh-7 and Hep3B cells after post-siSORT1 transfection, as assessed via propidium iodide (PI) staining and flow cytometry. Quantitative data for cells in different phases are shown on the right. **F** Flow cytometry analysis of Annexin V/PI-stained Huh-7 and Hep3B cells transfected with siSORT1 or control siRNA (NC). The quantification of apoptotic cells is shown on the right side. **G** Expression profiles of cell cycle-related proteins (p-Wee1, Cyclin B1, Cyclin D1, Cyclin D3, Cdc2, and Cdc25A) in Huh-7 and Hep3B cells after siSORT1 transfection, as detected via western blotting. The densitometric analysis of protein bands is shown on the right. **H** Western blot analysis of apoptosis-related proteins, including cleaved PARP, cleaved caspase 3, and cleaved caspase 9, in Huh-7 and Hep3B cells transfected with siSORT1 or NC. The densitometric quantification of bands is shown on the right. **I** Fluorescence microscopy images of Huh-7 and Hep3B cells stained with Hoechst33342 and PI after *SORT1* silencing. Apoptotic nuclei (yellow arrowheads) and damaged DNA (white arrowheads) are highlighted. The merged images provide a comprehensive view of the cell status with zoomed-in insets for clarity. Scale bars represent 100 µm. All experiments were repeated at least three times, and representative data are shown. Statistical significance is indicated by *p < 0.05, **p < 0.01, and ***p < 0.001. The analysis was performed using Welch’s *t*-test.

To gauge the functional implications of *SORT1* overexpression in HCC, we performed a series of in vitro assays using HCC and THLE-2 cells, a normal liver cell line. Quantitative real-time polymerase chain reaction (qRT-PCR) and western blot analyses corroborated the elevated *SORT1* expression in the majority of HCC-derived cell lines compared to that in THLE-2 cells (Supplementary Fig. 2A, B). Next, *SORT1* expression was reduced using RNA interference, and knockdown efficiency was verified in HCC cells at different time points (Supplementary Fig. 2C). Silencing *SORT1* using specific small interfering RNAs (siRNAs) in Huh-7 and Hep3B cells considerably reduced cellular proliferation. This reduction was evident from the diminished growth curves observed over 96 h as well as a marked decrease in cell viability (Fig. 3A, B). Furthermore, the colony-forming ability, an indicator of tumorigenic potential, was also reduced in the absence of *SORT1*, further emphasizing its pivotal role in HCC pathogenesis (Fig. 3C). *SORT1*-depleted cells also displayed compromised migratory potential in wound-healing assays, highlighting the importance of HCC cell migration (Fig. 3D).

Furthermore, propidium iodide (PI) staining and flow cytometric analysis, performed to understand the cell cycle dynamics, revealed noticeable alterations in cell cycle distribution following *SORT1* depletion. Specifically, a significant cell accumulation was observed in the G0/G1 phase, with a concomitant decrease in the S phase, suggesting G0/G1 cell cycle arrest in siSORT1-transfected cells (Fig. 3E). To elucidate the molecular mechanisms underlying cell cycle arrest, we assessed the expression profiles of key cell cycle regulators. A previous study showed that during the cell cycle, mitotic entry is regulated by Cdc2, which is activated and inhibited by cdc25A and p-Wee1, respectively [14]. Furthermore, flow cytometry with Annexin V/PI staining showed a marked increase in early and late apoptotic populations in siSORT1-transfected Huh-7 and Hep3B cells compared to that in control siRNA-treated cells (Fig. 3F). In addition, western blotting results indicated Cyclin B1, Cyclin D1, Cyclin D3, Cdc2, and Cdc25A downregulation, along with cell cycle inhibitor p-Wee1 upregulation in siSORT1-transfected Huh-7 and Hep3B cells, corroborating the cell cycle arrest at G0/G1 (Fig. 3G). Similarly, western blot analyses revealed elevated levels of key apoptotic markers, including cleaved poly ADP-ribose polymerase, cleaved caspase 3, and cleaved caspase 9, in both Huh-7 and Hep3B cells after post-*SORT1* knockdown (Fig. 3H). Fluorescence microscopy, post-Hoechst33342 and PI staining, performed to visually confirm these findings, revealed distinct nuclear morphological changes in siSORT1-transfected cells, including chromatin condensation and DNA fragmentation, the hallmark features of apoptosis (Fig. 3I). Collectively, these data highlight the instrumental role of *SORT1* in regulating HCC cell survival and proliferation.

### Silencing of *SORT1* inhibits HCC progression in animal models

To comprehensively assess the role of *SORT1* in HCC progression in vivo, we used both subcutaneous xenograft and orthotopic HCC mouse models to compare the development and progression of HCC in the *SORT1*-silenced (siSORT1) and negative control (NC) groups.

**Fig. 4:**
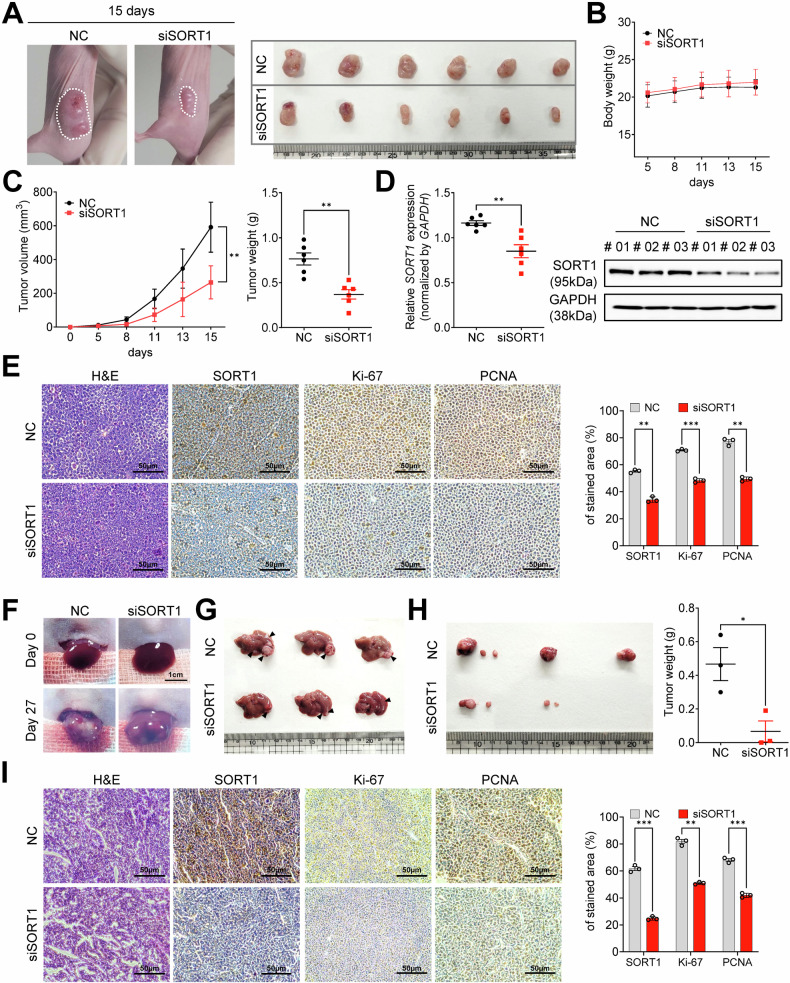
In vivo attenuation of hepatocellular carcinoma progression via *SORT1* silencing. **A** Representative images showing tumor progression on day 15 in mice after *SORT1* silencing, highlighting the reduced tumor size in the siSORT1 group compared to that in the NC group. **B** Body weight changes in mice over 15 d; NC vs. siSORT1 conditions. **C** Quantitative analysis of tumor growth over 15 d, highlighting a marked reduction in tumor size following *SORT1* silencing (left). Comparative assessment of tumor weights between NC and siSORT1 mice (right). **D** Relative *SORT1* mRNA expression in tumor tissues (left) and protein expression levels in NC versus siSORT1 conditions (right). **E** Histopathological examination of the tumors. Hematoxylin and eosin (H&E) staining showed the tumor architecture (leftmost). Subsequent panels display immunohistochemical staining for SORT1, Ki-67, and PCNA in both the NC and siSORT1 groups, with quantification of the stained areas on the right. **F** Visual representation of mouse liver on days 0 and 27 after siRNA treatment. **G** Gross anatomy of excised tumors from NC and siSORT1 mice. **H** Comparative display of metastatic nodules excised from the livers of the NC and siSORT1 groups (left). Quantitative analysis of the weights of these nodules indicated a decrease in metastatic potential upon *SORT1* silencing (right). **I** Tumor histological assessment post-metastasis: H&E staining of metastatic nodules (left), followed by immunohistochemical analyses for SORT1, Ki-67, and PCNA. The right panel shows the quantification of the stained regions, confirming the reduced metastatic potential and cell proliferation upon *SORT1* silencing. Scale bars represent 50 µm. All experiments were repeated at least three times, and representative data are shown. Statistical significance is indicated by *p < 0.05, **p < 0.01, and ***p < 0.001. The analysis was performed using Welch’s *t*-test.

In the subcutaneous xenograft HCC mouse model, visual observation on day 15 revealed a substantial reduction in tumor size in the siSORT1 group compared to that in the NC group (Fig. 4A). Moreover, both groups maintained consistent body weights throughout the study period, indicating that *SORT1* silencing did not adversely affect the overall mouse health (Fig. 4B). When monitoring tumor progression, the siSORT1 group exhibited a significantly slower growth curve than the NC group, further substantiating the tumor-suppressive role of *SORT1* silencing (Fig. 4C). Molecular validation through qRT-PCR and western blot analysis confirmed the significant downregulation of SORT1 in tumor tissues from the siSORT1 group, corroborating successful knockdown (Fig. 4D). In addition, histopathological examination of tumor sections from both groups using hematoxylin and eosin (H&E) staining provided insights into cellular morphology and organization. Immunohistochemical analyses highlighted the diminished *SORT1* expression and reduced staining of the proliferative markers Ki-67 and PCNA in the siSORT1 group, indicating a reduced cellular proliferation rate (Fig. 4E).

For the orthotopic HCC model, liver images from siSORT1-treated mice on day 27 showed a noticeable reduction in tumor occurrence compared to those from their NC counterparts (Fig. 4F). Gross anatomical examination of the extracted livers further confirmed smaller size and fewer tumors in the siSORT1 group (Fig. 4G, H). Further microscopic examination of these nodules after *SORT1* inhibition revealed reduced staining intensities for SORT1, Ki-67, and PCNA (Fig. 4I), indicating reduced cellular proliferation. Collectively, these findings underscore the pivotal role of *SORT1* in driving HCC progression and in effectively attenuating HCC progression in vivo through silencing.

### *SORT1* modulates angiogenesis and systemic metastasis by promoting epithelial-to-mesenchymal transition (EMT) in HCC

**Fig. 5:**
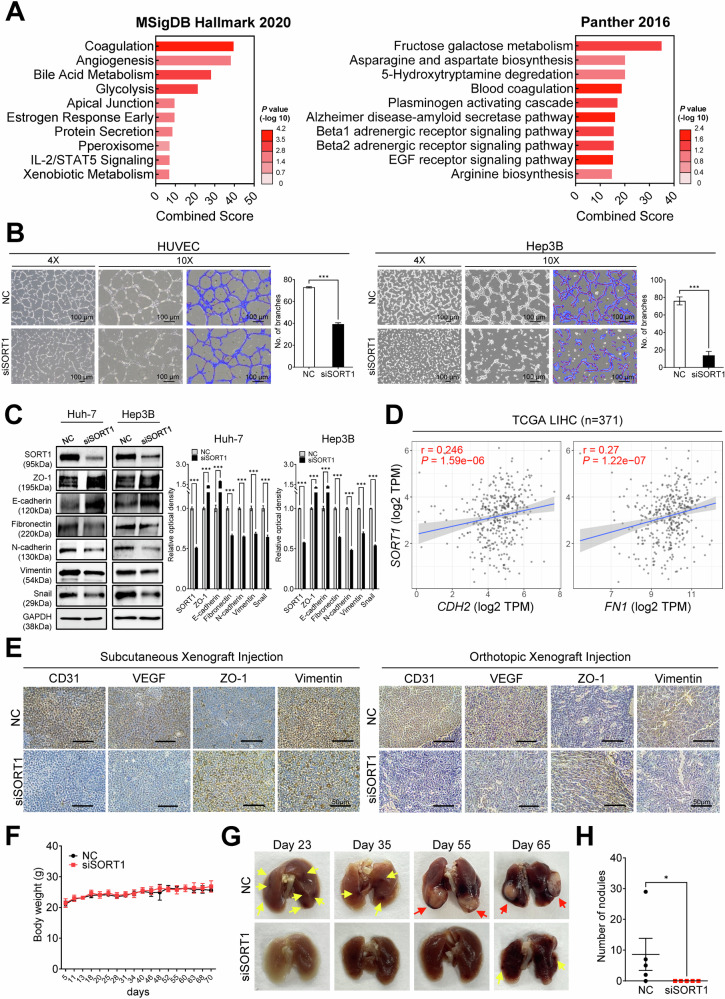
Functional impact of SORT1 on angiogenesis and systemic metastasis in HCC. **A** Pathway analysis showing the most significant pathways related to SORT1 from the MSigDB Hallmark 2020 and Panther 2016 databases sorted by combined score. The *p* value is represented by color intensity. **B** Tube-formation assay in NC or siSORT1 transfected HUVECs and Hep3B cells. Images are shown at 4× and 10× magnifications. A quantitative analysis of the total tube length is shown on the right. **C** Western blot analysis of various epithelial-to-mesenchymal transition (EMT) markers in NC- or siSORT1-treated Huh-7 and Hep3B cells. Densitometric quantification is shown on the right-hand side. **D** Scatter plots illustrating the correlation between *SORT1* and *CDH2* (N-cadherin) and *SORT1* and *FN1* (fibronectin) based on TCGA LIHC data. The correlation coefficients (r) and *p*-value are provided. **E** Immunohistochemical staining of CD31, VEGF, ZO-1, and vimentin in tumors from mice with subcutaneous and orthotopic xenograft injections of either NC or siSORT1-transfected cells. **F** Body weight of mice subjected to either NC or siSORT1 treatment over a specified period. **G** Representative images of lung tumor nodules in mice on days 23, 35, 55, and 65 post-injection with either NC or siSORT1-transfected cells. Yellow arrows indicate precancerous blood dots and red arrows indicate tumors. **H** Quantitative analysis of the number of nodules formed in the lungs of mice treated with NC or siSORT1. Scale bars represent 50 µm. All experiments were repeated at least thrice, and representative data are shown. Statistical significance is indicated by *p < 0.05, **p < 0.01, and ***p < 0.001. The analysis was performed using Welch’s *t*-test.

Pathway analysis using MSigDB Hallmark 2020 and Panther 2016 datasets revealed the potential involvement of *SORT1* in crucial cellular processes related to HCC, such as angiogenesis and coagulation (Fig. 5A). Factors involved in coagulation and angiogenesis are closely associated with EMT and tumor metastasis. To elucidate the mechanistic implications of *SORT1* in these processes, we performed a series of in vitro and in vivo experiments. First, we conducted a tube-formation assay using HUVECs (endothelial cells at the interface between the blood and the vessel wall) transfected with NC or siSORT1 to assess their effect on angiogenesis. Additionally, Hep3B cells with epithelial cell properties were analyzed for tube formation. The findings revealed a discernibly reduced tube-formation ability in the siSORT1 group compared to that in the control group (Fig. 5B). To further explore this molecular nexus, we measured the expression of essential EMT markers in Huh-7 and Hep3B cells. Following siSORT1 treatment, the expression of these markers was considerably altered, suggesting an EMT inhibition. Specifically, decreased expression of mesenchymal markers such as N-cadherin, vimentin, and fibronectin, coupled with the reinstatement of the epithelial marker E-cadherin, was evident (Fig. 5C). These findings were further supported by TCGA LIHC data, which demonstrated a significant correlation between the expression of *SORT1* and the EMT markers *CDH2* (N-cadherin) and *FN1* (fibronectin; Fig. 5D).

Our in vivo experiments revealed that tumors derived from *SORT1*-silenced HCC cells exhibited reduced staining of angiogenesis markers, including CD31 and VEGF, as well as and the EMT marker Vimentin, in both subcutaneous and orthotopic xenograft models. Conversely, ZO-1 staining was increased in the siSORT1 group compared to the NC group in both models (Fig. 5E). The decrease in CD31, VEGF, and Vimentin staining indicates reduced angiogenic and metastatic potential of SORT1-silenced cells, while the increase in ZO-1 suggests a shift towards an epithelial phenotype. Furthermore, the lung metastasis assay following tail vein injection of HCC cells treated with siSORT1 or NC revealed no significant difference in body weight between the two groups in the tail vein injection model (Fig. 5F). However, the siSORT1 group displayed fewer metastatic lung nodules on days 23, 35, 55, and 65 than the control group (Fig. 5G). Quantification revealed a significant reduction in the number of metastatic lung nodules in the siSORT1 group (Fig. 5H). These findings highlight that SORT1 is a key molecular player in modulating angiogenesis and EMT in HCC, thereby influencing tumor progression and systemic metastasis.

### SORT1 positively regulates Notch signaling activity and CD133 expression in HCC

We conducted an unbiased screening using both scRNA-seq and bulk RNA-seq data to identify top-ranked pathways and signaling events underlying the oncogenic role of *SORT1*. For HCC scRNA-seq data, we performed pseudo-bulk analysis using the AggregateExpression function in the Seurat package (v4.3.0). Samples were stratified based on aggregated *SORT1* expression and designated as *SORT1*^high^ (1st quartile), *SORT1*^mid^ (2nd and 3rd quartiles), and *SORT1*^low^ (4th quartile) groups. For bulk RNA-seq data, we stratified samples into *SORT1*^high^ and *SORT1*^low^ groups based on their normalized *SORT1* expression. Subsequently, we conducted differential expression analysis comparing *SORT1*^high^ and *SORT1*^low^ groups to investigate how differential expression of *SORT1* can affect HCC progression, including Notch signaling. The identified differentially expressed genes (DEGs) were ordered based on descending log2 fold change values and subjected to enrichment analysis using Gene Ontology (GO) and Gene Set Enrichment Analysis (GSEA) (Supplementary Fig. 3A, B).

**Fig. 6:**
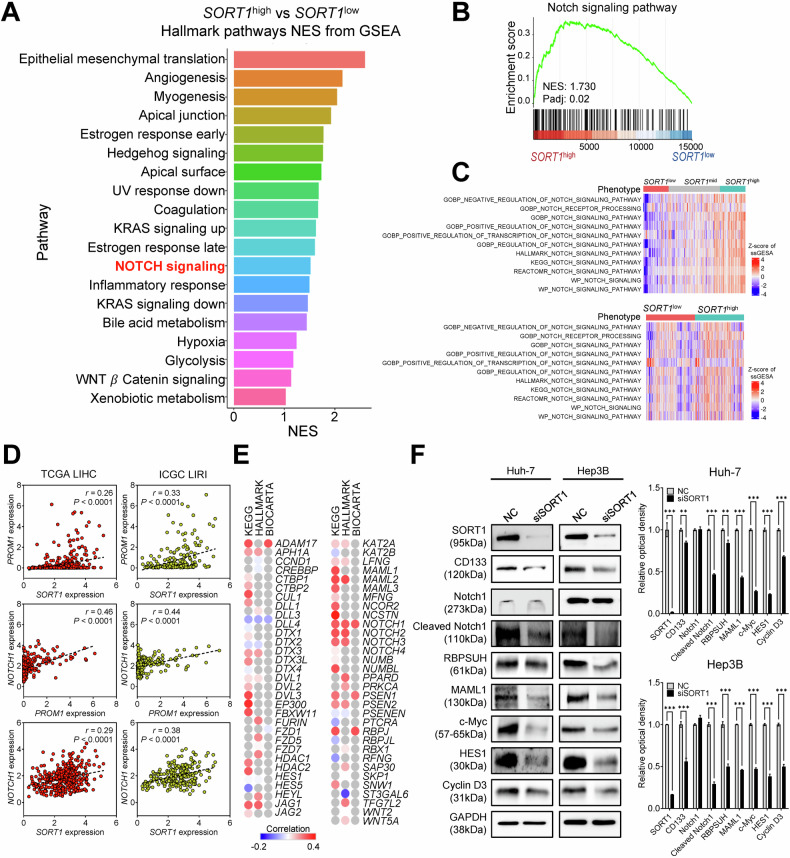
Correlation of SORT1 expression with CD133 and the Notch Signaling pathway in HCC. **A** GSEA hallmark pathways analysis identifying the Notch signaling pathway as one of the top-ranked pathways in SORT1^high^ samples. **B** GO enrichment analysis confirming the enrichment of Notch signaling-related gene sets in SORT1^high^ samples. **C** Wilcoxon rank-sum test results showing ssGSEA scores of each Notch signaling pathway in samples stratified by SORT1 expression (top: scRNA-seq; bottom: bulk RNA-seq data). **D** Scatter plots representing the positive correlation between *SORT1* and *PROM1* (CD133), *PROM1* and *NOTCH1*, and *SORT1* and *NOTCH1* expression in The Cancer Genome Atlas Liver Hepatocellular Carcinoma (TCGA LIHC) and ICGC LIRI datasets. Each dot represents a single sample. Pearson’s correlation coefficients (r) and *p* values are indicated for each plot. **E** Heatmap illustrating the correlation between *SORT1* and selected genes involved in the Notch signaling pathway derived from the KEGG, Hallmark, and BIOCARTA datasets. Genes were chosen based on their established role in the Notch pathway and their significant positive correlation with *SORT1* in TCGA LIHC dataset. The color intensities of the circles are proportional to the correlation coefficients. **F** Western blot analyses of Huh-7 and Hep3B cells treated with NC or siSORT1 showing the expression levels of Notch1, cleaved Notch1, and other pivotal Notch signaling molecules, along with CD133, in HCC cells. Statistical significance is indicated by **p < 0.01, ***p < 0.001. The analysis was performed using Welch’s *t*-test.

Notably, the Notch signaling pathway was identified as one of the top-ranked pathways in GSEA hallmark pathways analysis, in which related genes were highly expressed in the *SORT1*^high^ group (Fig. 6A). Moreover, we confirmed that GO gene sets related to the Notch signaling pathway were enriched in *SORT1*^high^ samples compared to *SORT1*^low^ samples (Fig. 6B).

For bulk RNA-seq data, while the Notch signaling pathway was not identified as a top-ranked pathway in GO enrichment analysis, GSEA identified several significant GO gene sets related to the Notch signaling pathway that were enriched in *SORT1*^high^ samples (Supplementary Fig. 3C).

Additionally, single-sample GSEA (ssGSEA) using normalized counts (for bulk RNA-seq) and aggregated expression (for scRNA-seq) for various Notch signaling-related gene sets revealed significant enrichment in *SORT1*^high^ samples, further supporting the involvement of the Notch signaling pathway. Wilcoxon rank-sum test results showing ssGSEA scores of each Notch signaling pathway in samples stratified by *SORT1* expression are shown below (Fig. 6C, top: scRNA-seq; bottom: bulk RNA-seq data). While the “GOBP negative regulation of Notch signaling pathway” gene set showed significant enrichment in scRNA-seq data, it did not show significant enrichment in bulk RNA-seq data. Importantly, the “GOBP positive regulation of Notch signaling pathway” gene set showed significant enrichment in SORT1^high^ samples in both scRNA-seq and bulk RNA-seq data.

We also conducted gene expression analyses using TCGA LIHC and International Cancer Genome Consortium Liver Cancer RIKEN (ICGC LIRI) data. Significant positive correlations were observed between *SORT1* and *PROM1, PROM1* and *NOTCH*, and *SORT1* and *NOTCH* expression in both cohorts (Fig. 6D). Additionally, most Notch-associated genes from the KEGG, Hallmark, and BIOCARTA datasets in TCGA LIHC were positively correlated with *SORT1* expression (Fig. 6E). Functional analyses to gain mechanistic insights demonstrated substantial downregulation of critical Notch signaling components in Huh-7 and Hep3B cells with silenced *SORT1*. The downregulated components included cleaved Notch1, RBPSUH, and MAML1 gene products associated with the activation of the Notch pathway, CD133, and other downstream molecules such as c-Myc and HES1 (Fig. 6F and Supplementary Fig. 3E). Additionally, we conducted RNA stability assays to investigate the impact of SORT1 knockdown on the mRNA stability of key Notch signaling components, including *NOTCH1*, *RBPJ*, *MAML1*, *MYC*, *HES1*, and *CCND3*. Our results showed no significant changes in the mRNA levels of these components upon SORT1 knockdown, suggesting that SORT1’s regulatory role may not affect mRNA stability (Supplementary Fig. 3D). Furthermore, we conducted co-immunoprecipitation (Co-IP) experiments to examine the interaction between CD133 and S2 complex proteins. The results demonstrated that CD133 does not directly bind to ADAM10 or ADAM17 (Supplementary Fig. 3F). In line with this, we found that controlling the expression of CD133 did not impact SORT1 expression or Notch signaling, indicating that CD133 is a downstream component regulated by SORT1 in HCC (Supplementary Fig. 3G, H). These findings suggest that SORT1 modulates the Notch pathway activity and regulates CD133 as a downstream HCC component.

### *SORT1* promoter hypomethylation at CpG Site cg16988986 drives *SORT1* overexpression: diagnostic and prognostic implications in HCC

**Fig. 7:**
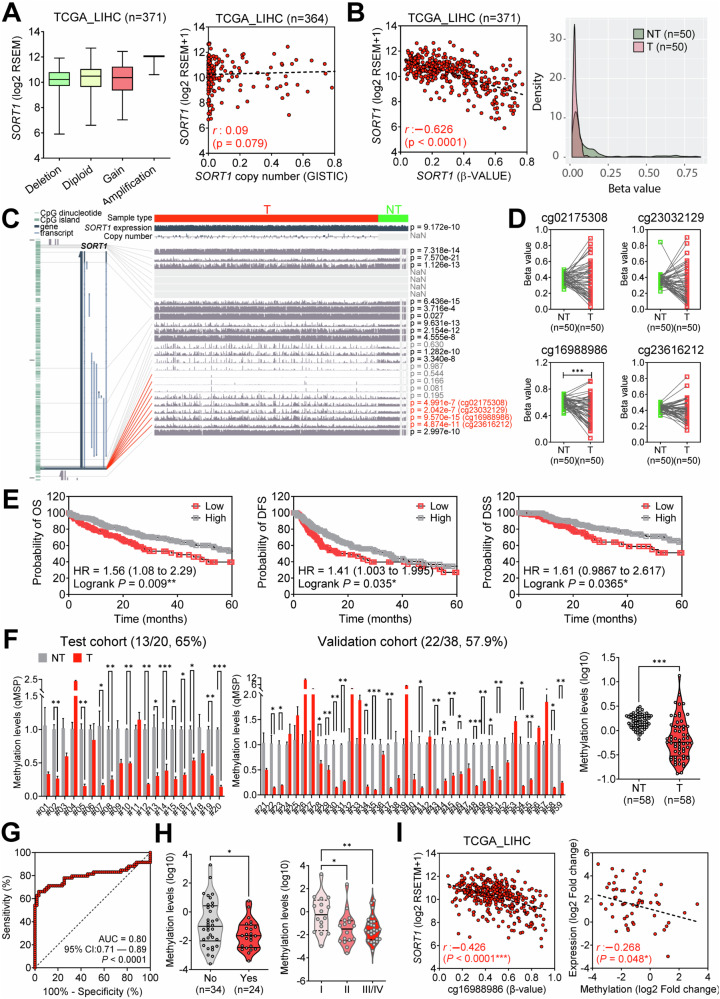
Comprehensive analysis of *SORT1* expression, genetic alteration, and clinical relevance in patients with hepatocellular carcinoma (HCC). **A** *SORT1* mRNA expression level based on gene copy number status from TCGA LIHC cohort (n = 371, left). Scatter plot illustrating the correlation between *SORT1* mRNA expression and copy number (GISTIC) in TCGA LIHC cohort (n = 364, right). **B** Scatter plot illustrating the correlation between *SORT1* mRNA expression and methylation in TCGA LIHC cohort (n = 371, left). Density plot displaying the methylation beta values for the (NT) and tumor (T) groups from the same cohort (right). **C** Genome-wide association between *SORT1* copy number variations and differential mRNA expression in HCC tumor tissues. The red line indicates the 5′ promoter region corresponding to *SORT1*. **D** Dot plots displaying differential methylation levels for four significant CpG sites related to SORT1 between non-tumoral (NT) and tumoral (T) liver tissues, with significance values presented for each site. **E** Kaplan–Meier survival curves comparing overall survival (OS) and disease-free survival (DFS) between HCC patients with high and low *SORT1* expression from The Cancer Genome Atlas (TCGA) database, accompanied by hazard ratio (HR) and log-rank *p* values. **F** Methylation levels at multiple CpG sites of *SORT1* in non-tumoral (NT) and tumoral (T) liver tissues, assessed in both the test (left) and validation cohorts (middle) from Ajou University Hospital. Violin plot of *SORT1* methylation in non-tumor (NT) and tumor (T) liver tissues in the total cohort from Ajou University Hospital (right). **G** Receiver operating characteristic (ROC) curve assessing the diagnostic performance of *SORT1* methylation in differentiating T from NT tissues. **H** Violin plot of *SORT1* methylation based on the vascular invasion status (left) and HCC stage (right). **I** Scatter plot depicting the inverse correlation between *SORT1* expression and its methylation level in The Cancer Genome Atlas Liver Hepatocellular Carcinoma (TCGA LIHC) and AJOU_HCC cohorts. Statistical significance is indicated by *p < 0.05, **p < 0.01, and ***p < 0.001. The analysis was performed using Student’s *t*-test and Welch’s *t*-test.

To elucidate the epigenetic mechanisms contributing to *SORT1* overexpression in HCC, we performed a comprehensive analysis utilizing data from TCGA LIHC cohort. This analysis investigated the prevalence of both SORT1 mutations and copy number variations (CNVs). As shown in Supplementary Fig. 4A, *SORT1* mutations and amplifications occurred at very low rates, with frequencies of 0.27% and 0.81%, respectively, indicating their rarity in HCC. This result was consistent with those of the other datasets (Supplementary Fig. 4B). Comparison of *SORT1* mRNA expression according to copy number status revealed no significant differences (Fig. 7A, left panel). Additionally, the correlation between *SORT1* mRNA expression and copy number was not significant (Pearson’s *r* = 0.09, *p* = 0.079; Fig. 7A, right panel). In contrast, a significant inverse relationship was observed between *SORT1* mRNA expression and methylation status (Fig. 7B, left panel), indicating that methylation status plays a central role in the regulation of *SORT1* expression. This distinct difference in the methylation patterns between non-tumor (NT) and tumor (T) groups was further validated using a density plot (Fig. 7B, right panel). These results suggest that *SORT1* overexpression in HCC primarily arises from hypomethylation, rather than from mutations or CNVs.

Next, we sought to identify the CpG islands within the 5’ promoter region of *SORT1* associated with its mRNA expression using MEXPRESS [15]. Among the various CpG sites linked to *SORT1*, four (cg02175308, cg23032129, cg16988986, and cg23616212) exhibited significant differences in methylation levels between non-tumor and tumor liver tissues (Fig. 7C). Among these four CpG sites, cg16988986 was significantly hypomethylated in tumor samples compared to that in non-tumor tissues in TCGA LIHC dataset (Fig. 7D). Furthermore, in TCGA DB, patients with a hypomethylated cg16988986 site demonstrated a significantly poor prognosis, with a lower overall and disease-free survival (Fig. 7E).

Therefore, we assessed the cg16988986 methylation levels in both non-tumor and tumor liver tissue samples from the AJOU_HCC cohort. In the test cohort, 65% of the liver cancer tissues displayed hypomethylation at this site (Fig. 7F, left panel), whereas 57.9% exhibited cg16988986 hypomethylation in the validation cohort (Fig. 7F, middle panel). When analyzing methylation levels across all 58 patients, tumor tissues consistently showed significantly reduced methylation compared to their non-tumor counterparts (Fig. 7F, right panel). A receiver operating characteristic (ROC) curve analysis of *SORT1* methylation at cg16988986 showed an area under the curve (AUC) of 0.8 (95% confidence interval [CI]: 0.71–0.89), suggesting its potential utility in distinguishing tumor tissues from non-tumor tissues (Fig. 7G). Furthermore, *SORT1* methylation status at cg16988986 exhibited significant differences according to the vascular invasion status (Fig. 7H, left panel) and tumor stage (Fig. 3H, right panel). Additionally, correlation analyses revealed a significant inverse correlation between *SORT1* methylation status at cg16988986 and *SORT1* mRNA expression in both TCGA LIHC and AJOU_HCC cohorts (Fig. 7I). To further validate the role of DNA methylation in regulating *SORT1* expression, we treated HCC cells with the methylation inhibitor 5-aza-2’-deoxycytidine (5-aza) at a concentration of 5 μM for 48 h. The results showed that 5-aza treatment significantly increased SORT1 expression (both mRNA and protein levels) and decreased methylation levels at the *SORT1* promoter (Supplementary Fig. 5A–C). This finding highlights the crucial role of DNA methylation in the regulation of *SORT1* expression and supports the potential diagnostic and prognostic utility of assessing *SORT1* methylation status in HCC.

## Discussion

This study aimed to identify the potential oncogenes in HCC and gain a deeper understanding of their roles in disease progression. The findings confirm that *SORT1* is a prominent candidate oncogene that contributes to HCC development and progression. Previous studies have indicated that *SORT1* is overexpressed in cancer tissues and plays an oncogenic role in various cancers, including HCC [16–20]. In contrast to studies that primarily relied on TCGA LIHC or one or two additional publicly available datasets to investigate *SORT1* expression in HCC, our study adopted a more comprehensive approach. We conducted a thorough analysis using eight distinct omics datasets to identify *SORT1* as a potential oncogenic candidate in HCC and validated SORT1 expression in an HCC cohort. Additionally, our study made a significant advancement by demonstrating *SORT1* upregulation in HCC cells at the single-cell level, in comparison to non-malignant hepatocytes, which was subsequently confirmed through meticulous analysis of single-cell datasets and spatial transcriptomic data using resources such as GepLiver and Mendeley DB.

Furthermore, through extensive in vivo and in vitro experiments, we confirmed that silencing *SORT1* effectively suppressed metastasis and angiogenesis in HCC. These findings reveal that the Notch pathway and CD133 are a crucial downstream oncogenic pathway influenced by SORT1. Furthermore, this study demonstrated the epigenetic mechanisms governing *SORT1* expression regulation, highlighting the significant inverse correlation between *SORT1* expression and cg16988986 methylation status. These comprehensive findings set our research apart from previous studies, which provided only limited information regarding the role of *SORT1* in HCC.

In this study, we revealed the potential role of *SORT1* in promoting angiogenesis and EMT in HCC cells. Angiogenesis plays a crucial role in cancer progression, and SORT1 has been reported to have a significant impact on cancer progression by regulating angiogenesis in several cancer types [7, 21]. Given that HCC is known for its hypervascularity and anti-angiogenic drugs such as bevacizumab are used as first-line treatments for HCC in combination with atezolizumab [22], *SORT1* may also act as a promising new therapeutic target for HCC as an anti-angiogenic agent with or without an immune modulator. Additionally, a lung metastasis assay revealed a marked reduction in the number of metastatic foci in animal models with silencing *SORT1* expression. EMT is a critical mechanism in cancer metastasis [23], and we demonstrated the regulation of EMT-related proteins according to *SORT1* expression status. In this functional study, we revealed that the oncogenic effects of *SORT1* are mediated through the activation of the Notch signaling pathway, and increased CD133 expression.

CD133, a recognized cancer stem cell (CSC) marker, has garnered increasing attention in cancer research [24]. CSCs play a pivotal role in tumor initiation, progression, and resistance to chemotherapy, making them potential targets for innovative cancer therapies [25]. Notch signaling is essential for various cellular processes, including differentiation, proliferation, and apoptosis [26]. Dysregulation of this pathway is implicated in numerous cancers, including HCC [27, 28]. In this study, we identified *SORT1* as a critical Notch signaling pathway and CD133 regulator in HCC. Our findings reveal that SORT1 enhances the stability of key Notch signaling components, specifically cleaved Notch1, thereby promoting the activation of the Notch pathway. This stabilization mechanism highlights SORT1’s role in facilitating downstream Notch signaling processes. These findings demonstrate the pivotal role of *SORT1* in HCC and shed light on our understanding of HCC and its potential therapeutic targets.

Moreover, we explored the epigenetic mechanisms governing *SORT1* expression. These findings demonstrated that *SORT1* expression was not associated with CNV or single nucleotide polymorphisms (SNPs); instead, hypomethylation at the *SORT1* promoter region was significantly correlated with *SORT1* overexpression. This correlation observed in TCGA data was validated using our real clinical data. Furthermore, the methylation status of a specific *SORT1* promoter region, cg16988986, not only showed potential as a diagnostic marker to differentiate HCC tissues from non-tumor tissues but also exhibited a significant correlation with patient prognosis, indicating its utility as a prognostic biomarker.

Our study has few limitations. First, although we showed that SORT1 regulates the Notch signaling pathway in HCC, we did not explore the detailed mechanisms by which SORT1 activates the Notch signaling pathway. Given the known cellular functions of SORT1, it may activate the Notch pathway through various mechanisms such as enhancing ligand-receptor interactions, modulating receptor localization, influencing receptor cleavage, and crosstalk with other signaling pathways. Further studies are required to elucidate the underlying mechanisms. Next, siRNA was used to silence *SORT1* expression. To further advance this research and develop *SORT1* as a novel therapeutic target, future studies should explore more clinically applicable approaches to regulate *SORT1*, such as the use of peptides or other suitable formulations.

In summary, our study highlights that SORT1 promotes proliferation, invasion, angiogenesis, and systemic metastasis in HCC through the activation of the Notch signaling pathway. The key mechanism regulating *SORT1* expression, cg16988986 hypomethylation, is significantly associated with poor prognosis in patients with HCC. These findings demonstrate that *SORT1* holds promise as a novel therapeutic target for HCC. This discovery paves the way for future treatments and applications; nevertheless, further research is required for practical development.

## Materials and methods

### Patients and specimens

In this study, HCC and the corresponding adjacent non-cancerous tissues were procured from 86 patients with HCC who underwent hepatectomy at the Ajou University Hospital, Suwon, South Korea. These samples were sourced from the Biobank of the Ajou University Hospital, a member of the Korea Biobank Network (http://www.kbn.re.kr/kbn/main.do). Supplementary Table 3 shows detailed patient demographics and clinical attributes. We named this HCC cohort the AJOU_HCC cohort.

### Identification of HCC driver gene signatures

To identify key driver genes in HCC, we conducted a comprehensive analysis of three distinct microarray datasets associated with various HCC stages. These datasets, GSE6764, GSE54238, and GSE89377, were obtained from the Gene Expression Omnibus (GEO) database of the National Center for Biotechnology Information (NCBI). Specifically, GSE6764 encompasses samples from 10 normal livers (NL), 13 liver cirrhosis (LC), 17 dysplastic nodules (DN), 18 early HCC (eHCC), and 17 advanced HCC (aHCC). GSE54238 included 10 NL, 10 chronic hepatitis (CH), 10 LC, 13 eHCC, and 13 aHCC samples, whereas GSE89377 included 13 NL, 20 CH, 12 LC, 22 DN, 14 eHCC, and 26 aHCC samples. From these datasets, we identified genes exhibiting marked differential expression in HCC relative to non-tumor (NT) samples by applying the criteria of *P*-adj < 0.05 and an absolute fold change greater than 1.5. Serial pattern analysis was performed using the CLICK algorithm [29]. We identified common genes across five RNA-seq datasets (The Cancer Genome Atlas, TCGA; International Cancer Genome Consortium, ICGC; GSE77314, GSE114564, and GSE124535) and validated the *SORT1* expression across four multi-stage HCC datasets. A schematic representation of the analysis is shown in Fig. 1A.

### Bulk and spatial transcriptomics data analysis

To analyze the bulk RNA expression of *SORT1* across tumor tissues with different phenotypes, we downloaded expression data (Transcripts Per Million; TPMs) from the GepLiver database (DB) [11]. Spatial transcriptomic data were downloaded from Mendeley (skrx2fz79n) software. Data analysis was performed using the R software (v.4.2.3). A heatmap was plotted using the Complex Heatmap package (v.2.14). Box and bar plots were generated using ggplot2 (version 3.4.3) and ggparl (v.0.0.1) packages. Spatial transcriptomic data were analyzed using the Seurat package (v.4.3.0) [30]. For bulk-level RNA-seq analysis, *SORT1* expression in each group was tested for normality using the Shapiro–Wilk normality test. Non-normally distributed groups were compared using the Wilcoxon rank-sum or Kruskal–Wallis rank-sum test and Dunn’s test. Statistical significance was set at *p* < 0.05.

### Single-cell RNA-seq data analysis

Single-cell RNA-seq (scRNA-seq) data were downloaded from GepLiver DB [11], comprising a total of 17 scRNA-seq datasets. For downstream analysis, samples from HCC, adjacent HCC (ADJ_HCC), and normal tissues were selected. Single-cell Atlas in Liver Cancer (scAtlasLC) data were obtained from the NCBI for Biotechnology Information Gene Expression Omnibus (GSE151530) [13]. Cell type annotations were provided by the authors of the original papers and encompassed both malignant and non-malignant hepatocytes classified using inferCNV [31]. To verify the presence of *SORT1* in HCC, we extracted a count matrix for *SORT1* using the Seurat package (version 4.0.0) in R (version 4.3.1) [30]. Any instances with a count of 0 were categorized as ‘*SORT1* negative,’ while those with a count of 1 or more were considered ‘*SORT1* positive.’ The proportion of *SORT1*-positive cells was determined by dividing the number of *SORT1*-positive cells by the total number of cells within each phenotype. *SORT1* counts for each cell were visualized using ggplot2 (v.3.4.3), which displays *SORT1* expression according to the phenotype [32]. Group comparisons were conducted using Student’s *t*-test with the ggpubr package (v.0.6.0) [33], and statistical significance was set at *p* < 0.05.

### Cell culture and transfection

Human HCC cell lines Huh-7, Hep3B, PLC/PRF/5, SNU368, SNU398, SNU423, SNU449, and SNU475 were obtained from the Korean Cell Line Bank (KCLB, Seoul, South Korea). The THLE-2 normal liver cell line was obtained from the American Type Culture Collection (ATCC, Manassas, VA, USA). HCC cell lines were cultured in Roswell Park Memorial Institute-1640 (RPMI-1640) or Dulbecco’s modified Eagle’s medium (DMEM) supplemented with 10% fetal bovine serum (FBS; Invitrogen, Waltham, MA, USA) and 100 U/mL penicillin-streptomycin (GenDEPOT, Barker, TX, USA). THLE-2 cells were maintained in a bronchial epithelial cell growth medium (Lonza, Walkersville, MD, USA) supplemented with 10% FBS, 5 ng/mL epidermal growth factor (Sigma-Aldrich, St. Louis, MO, USA), 70 ng/mL phosphoethanolamine (Sigma-Aldrich), and appropriate antibiotics. All cells were housed at 37 °C in a humidified incubator under a 5% CO_2_ atmosphere. For transfection, cells were seeded in 60 mm^2^ dishes and allowed to adhere overnight. Upon reaching 30–40% confluence, the cells were transfected with small interfering RNA (siRNA) and SORT1 or CD133 expressing vectors using Lipofectamine 2000 transfection reagent (Invitrogen), following the manufacturer’s protocol. The SORT1 or CD133 expressing vectors were purchased from VectorBuilder (Chicago, IL, USA).

### RNA isolation and quantitative real-time polymerase chain reaction (qRT-PCR) analysis

Total RNA was isolated from tissues and cells using the TRIzol reagent (Qiagen, Hilden, Germany). For cDNA synthesis, 500 ng isolated RNA was reverse-transcribed in a 10 μL reaction volume with the 5X PrimeScript™ RT Master Mix (Takara Bio, Shiga, Japan). qRT-PCR reactions incorporated the synthesized cDNA and were subjected to 40 amplification cycles: 95 °C for 15 s, 58–60 °C for 34 s, and 72 °C for 30 s. This was followed by a dissociation phase: 95 °C for 10 s, 65 °C for 5 s, and 95 °C for 5 s. Amplification was performed using the amfiSure qGreen Q-PCR Master Mix (GenDEPOT) and monitored using a CFX Connect Real-Time PCR Detection System (Bio-Rad Laboratories, Hercules, CA, USA). Gene expression levels, relative to glyceraldehyde 3-phosphate dehydrogenase (*GAPDH*), were computed using the 2^−ΔΔ^Ct method. The primers used for *SORT1* were as follows: forward, 5′-AGTTTCAGTGACCCACGTCAG-3′ and reverse, 5′-AGTAGGTCAGGTAACAAAGTCCAGT-3′; *GAPDH* forward, 5′-AGTATGACAACAGCCTCAAG-3′ and reverse, 5′-TCATGAGTCCTTCCACGATA-3′. The assays were conducted in triplicate.

### Western blotting

Tissue or cell proteins were extracted using T-PER buffer or RIPA buffer supplemented with Halt™ Protease Inhibitor Cocktail (Thermo Fisher Scientific, Waltham, MA, USA). Protein concentrations were determined using the Pierce BCA Protein Assay Kit (Thermo Fisher Scientific). Subsequently, 5–10 μg protein lysates were resolved on sodium dodecyl sulfate-polyacrylamide gels and then electroblotted onto polyvinylidene difluoride membranes (Millipore, Billerica, MA, USA). The membranes were blocked with 5% skim milk for 1 h at 22–25 °C. After blocking, the sections were incubated overnight at 4 °C with specific primary antibodies, followed by incubation with the appropriate HRP-conjugated secondary antibodies. Supplementary Table 4 lists the details of the antibodies used. Also, full and uncropped western blot images are uploaded as Supplemental Material.

### Wound-healing assay

First, cells at a density of 1.5 × 10^6^ were seeded in 60 mm dishes and transfected with NC or siSORT1. After transfection, cells (1.5 × 10^6^) were reseeded in 6-well plates. Once the cells reached confluency, a scratch was made using a sterile 200 µL micropipette tip to create a wound. Images of the wounds at 0 and 48 h post-scratch were captured using a CKX53 microscope (Olympus). The assay was performed in triplicate, and the results are expressed as the percentage of wound closure.

### Tube-formation assay

For the tube-formation assay, 24-well plates were coated with 150 µL Matrigel Basement Membrane Matrix (BD Biosciences) and allowed to set at 37 °C for 1 h. Subsequently, 1.5 × 10^4^ HUVECs or Hep3B cells transfected with NC or siSORT1 were resuspended and added to the Matrigel-coated wells. The plates were then incubated at 37 °C in a humidified environment. Following incubation, the branching patterns of the tubular structures were quantitatively analyzed using the MyWim image analysis platform (https://mywim.wimasis.com).

### In vivo assays

Five-week-old female BALB/c nude mice (ORIENT BIO Inc., Seongnam, Korea) and six-week-old athymic female nude mice (Koatech, Pyeongtaek, South Korea) were used in this study. The mice were housed under specific pathogen-free conditions in individually ventilated cages. The housing environment was maintained at a temperature of 22 ± 2 °C with a 12-h light/dark cycle and humidity controlled at 50 ± 10%. The mice were given a week to acclimatize to these conditions before the commencement of the experiments.

To establish a subcutaneous xenograft model, Huh-7 cells (5 × 10^5^) transfected with siSORT1 or NC were mixed with Matrigel (Corning, NY, USA) and serum-free Dulbecco’s modified Eagle’s medium (GenDEPOT, Barker, TX, USA) and subcutaneously injected into the flanks of BALB/c nude mice (n = 6). Tumor growth was assessed three times weekly for two weeks using a digital caliper, and tumor volume was calculated as 0.52 × length × width^2^. On day 15, the mice were humanely euthanized by carbon dioxide asphyxiation per the standard ethical guidelines for animal research. Following euthanasia, tumors were extracted for weight measurement, and tissue samples were processed for RNA/protein extraction and histological evaluation.

To develop an orthotopic model, Huh-7 liver cancer cells transfected with either siRNA targeting SORT1 (siSORT1) or negative control siRNA (NC; see Supplementary Materials) were prepared in a solution of 5 × 10^5^ cells in Matrigel and serum-free Dulbecco’s modified Eagle’s medium. This mixture was orthotopically injected into the livers of five-week-old BALB/c nude mice (n = 3), which required a small surgical procedure under anesthesia. Ketamine was used as an anesthetic during orthopedic surgery in this mouse model. Postoperatively, the mice were monitored and tumor development was assessed regularly using noninvasive imaging.

Using a tail vein injection model, Hepa1-6 cells post-transfected with either siSORT1 or NC (2 × 10^6^ cells) were injected into athymic nude mice (n = 5). After 23, 35, 55, and 65 days, the mice in each group were sacrificed, and lung tissues were examined for metastatic nodules to evaluate lung metastasis.

### Immunohistochemistry (IHC)

Tumor specimens were extracted, fixed in 10% neutral-buffered formalin, embedded in paraffin, and cut into 5-μm thick sections. The sections were divided into two halves; one was used for H&E staining and the other for IHC. For IHC, sections were deparaffinized in xylene and rehydrated using graded alcohol. Subsequently, the sections were exposed to primary antibodies and incubated overnight at 4 °C. After incubation, the sections were rinsed three times and incubated with secondary antibodies for 1 h. Subsequently, peroxidase substrate was added until the desired staining intensity was achieved. Supplementary Table S4 lists the specific details of the antibodies used.

### Exploration of *SORT1* alterations using cBioPortal and comparative analysis

We performed a comprehensive analysis of *SORT1* alterations, identified CNVs, mutations, and mRNA expression levels, and explored their correlation with relevant clinical data using cBioPortal (https://www.cbioportal.org/). Within the portal, we focused on “TCGA PanCancer Atlas Studies”, which included data from 10,967 samples spanning 32 different cancer types.

### Quantitative methylation-specific polymerase chain reaction (qMSP)

For the qMSP analysis, primers specific for either methylated or unmethylated DNA were designed using MethPrimer 2.0 (http://www.urogene.org/cgi-bin/methprimer/methprimer.cgi). We amplified bisulfite-treated genomic DNA under the following cycling conditions: 40 cycles of denaturation at 98 °C for 10 s, annealing at 58 °C for 40 s, and extension at 72 °C for 30 s. qMSP was performed using the amfiSure qGreen Q-PCR Master Mix (GenDEPOT), and the reactions were monitored in real time using the CFX Connect Real-Time PCR Detection System (Bio-Rad Laboratories). The sequences of the primers used were as follows: *SORT1_Methyl*: forward, 5′-GTTTTGTTGTAAGAAGGTGAATGC-3′; reverse, 5′-AAAAAAATAAAAAAAACCAAACGTA-3′; *SORT1_Unmethyl*: forward, 5′-GTTTTGTTGTAAGAAGGTGAATGTG-3′; and reverse, 5′-AAAAAAATAAAAAAAACCAAACATA-3′. Relative DNA methylation levels were determined by computing the difference between the Ct values of methylated and unmethylated PCR products. Each measurement was performed in triplicate.

### Statistical analysis

Statistical analyses were performed using GraphPad Prism (version 9.0; GraphPad Software, San Diego, CA, USA), except for ROC analysis, which was conducted using the IBM SPSS software (IBM SPSS Statistics for Windows, version 22.0, released 2013, IBM, USA). The paired Student’s *t*-test was used to compare tumor and non-tumor tissues within the same patients, while the unpaired Welch’s *t*-test was used to evaluate all other groups. For evaluations involving more than two groups, one-way analysis of variance (ANOVA) followed by Tukey’s post-hoc analysis was used. Kaplan–Meier survival curves were generated, and significance was assessed using the log-rank test. The analytical approach involved the generation of ROC curves to evaluate the diagnostic accuracy of the biomarkers under investigation. These curves were used to assess the sensitivity and specificity of the biomarkers across various threshold values. Additionally, the area under the ROC curve (AUROC) was calculated to provide a quantitative measure of the overall diagnostic effectiveness. This analysis was complemented by 95% CIs for AUROC, offering a statistical range within which the true AUC was expected to lie, thereby indicating the precision of our assessment. All experiments were repeated at least three times, and a *p* < 0.05 was considered statistically significant.

### Supplementary information

Supplementary information

Original Data

## Data Availability

RNA-sequencing datasets generated in this study are deposited in the Gene Expression Omnibus database under accession code GSE6764, GSE54238, GSE89377, and GSE151530.

## References

[1] Yang JD, Hainaut P, Gores GJ, Amadou A, Plymoth A, Roberts LR. A global view of hepatocellular carcinoma: trends, risk, prevention and management. Nat Rev Gastroenterol Hepatol. 2019;16:589–604. 31439937 10.1038/s41575-019-0186-y PMC6813818

[2] Yang C, Zhang H, Zhang L, Zhu AX, Bernards R, Qin W, et al. Evolving therapeutic landscape of advanced hepatocellular carcinoma. Nat Rev Gastroenterol Hepatol. 2023;20:203–22. 36369487 10.1038/s41575-022-00704-9

[3] Huang A, Yang XR, Chung WY, Dennison AR, Zhou J. Targeted therapy for hepatocellular carcinoma. Signal Transduct Target Ther. 2020;5:146. 32782275 10.1038/s41392-020-00264-x PMC7419547

[4] Kjolby M, Andersen OM, Breiderhoff T, Fjorback AW, Pedersen KM, Madsen P, et al. Sort1, encoded by the cardiovascular risk locus 1p13.3, is a regulator of hepatic lipoprotein export. Cell Metab. 2010;12:213–23. 20816088 10.1016/j.cmet.2010.08.006

[5] Mitok KA, Keller MP, Attie AD. Sorting through the extensive and confusing roles of sortilin in metabolic disease. J Lipid Res. 2022;63:100243. 35724703 10.1016/j.jlr.2022.100243 PMC9356209

[6] Kjolby M, Nielsen MS, Petersen CM. Sortilin, encoded by the cardiovascular risk gene SORT1, and its suggested functions in cardiovascular disease. Curr Atheroscler Rep. 2015;17:496. 25702058 10.1007/s11883-015-0496-7

[7] Charfi C, Demeule M, Currie JC, Larocque A, Zgheib A, Danalache BA, et al. New peptide-drug conjugates for precise targeting of SORT1-mediated vasculogenic mimicry in the tumor microenvironment of TNBC-derived MDA-MB-231 breast and ovarian ES-2 clear cell carcinoma cells. Front Oncol. 2021;11:760787. 34751242 10.3389/fonc.2021.760787 PMC8571021

[8] Liu Y, Zhang J, Chen Y, Sohel H, Ke X, Chen J, et al. The correlation and role analysis of COL4A1 and COL4A2 in hepatocarcinogenesis. Aging. 2020;12:204–23. 31905170 10.18632/aging.102610 PMC6977693

[9] Zhang H, Wang Y, Ding H. COL4A1, negatively regulated by XPD and miR-29a-3p, promotes cell proliferation, migration, invasion and epithelial-mesenchymal transition in liver cancer cells. Clin Transl Oncol. 2021;23:2078–89. 33891266 10.1007/s12094-021-02611-y

[10] Wang T, Jin H, Hu J, Li X, Ruan H, Xu H, et al. COL4A1 promotes the growth and metastasis of hepatocellular carcinoma cells by activating FAK-Src signaling. J Exp Clin Cancer Res. 2020;39:148. 32746865 10.1186/s13046-020-01650-7 PMC7398077

[11] Li Z, Zhang H, Li Q, Feng W, Jia X, Zhou R, et al. GepLiver: an integrative liver expression atlas spanning developmental stages and liver disease phases. Sci Data. 2023;10:376. 37301898 10.1038/s41597-023-02257-1 PMC10257690

[12] Xun Z. Integrating single-cell and spatial transcriptomics to elucidate the cross-talk of SPP1+ macrophage and cancer associated fibroblast in HCC with immune excluded microenvironment. Mendeley Data. 2022. 10.17632/skrx2fz79n.1.

[13] Ma L, Wang L, Khatib SA, Chang CW, Heinrich S, Dominguez DA, et al. Single-cell atlas of tumor cell evolution in response to therapy in hepatocellular carcinoma and intrahepatic cholangiocarcinoma. J Hepatol. 2021;75:1397–408. 34216724 10.1016/j.jhep.2021.06.028 PMC8604764

[14] Watanabe N, Broome M, Hunter T. Regulation of the human WEE1Hu CDK tyrosine 15-kinase during the cell cycle. EMBO J. 1995;14:1878–91. 7743995 10.1002/j.1460-2075.1995.tb07180.x PMC398287

[15] Koch A, De Meyer T, Jeschke J, Van Criekinge W. MEXPRESS: visualizing expression, DNA methylation and clinical TCGA data. BMC Genom. 2015;16:636. 10.1186/s12864-015-1847-z PMC4549898 26306699

[16] Gao Y, Li Y, Song Z, Jin Z, Li X, Yuan C. Sortilin 1 promotes hepatocellular carcinoma cell proliferation and migration by regulating immune cell infiltration. J Oncol. 2022;2022:6509028. 35847356 10.1155/2022/6509028 PMC9286884

[17] Lin M, Zhu M, Ge T, Lu N, Fu X, Chang J. Prognostic potential and mechanism of SORT1 and its co‐expressed genes in hepatocellular carcinoma based on integrative analysis of multiple database. Precis Med Sci. 2022;11:161–73. 10.1002/prm2.12084

[18] Demeule M, Charfi C, Currie JC, Zgheib A, Danalache BA, Beliveau R, et al. The TH1902 docetaxel peptide-drug conjugate inhibits xenografts growth of human SORT1-positive ovarian and triple-negative breast cancer stem-like cells. Pharmaceutics. 2022;14:1910. 36145658 10.3390/pharmaceutics14091910 PMC9503230

[19] Liang M, Yao W, Shi B, Zhu X, Cai R, Yu Z, et al. Circular RNA hsa_circ_0110389 promotes gastric cancer progression through upregulating SORT1 via sponging miR-127-5p and miR-136-5p. Cell Death Dis. 2021;12:639. 34162830 10.1038/s41419-021-03903-5 PMC8222372

[20] Blondy S, Talbot H, Saada S, Christou N, Battu S, Pannequin J, et al. Overexpression of sortilin is associated with 5-FU resistance and poor prognosis in colorectal cancer. J Cell Mol Med. 2021;25:47–60. 33325631 10.1111/jcmm.15752 PMC7810928

[21] Fan X, Khaki L, Zhu TS, Soules ME, Talsma CE, Gul N, et al. NOTCH pathway blockade depletes CD133-positive glioblastoma cells and inhibits growth of tumor neurospheres and xenografts. Stem Cells. 2010;28:5–16. 19904829 10.1002/stem.254 PMC2878196

[22] Bruix J, Chan SL, Galle PR, Rimassa L, Sangro B. Systemic treatment of hepatocellular carcinoma: an EASL position paper. J Hepatol. 2021;75:960–74. 34256065 10.1016/j.jhep.2021.07.004

[23] Tiwari N, Gheldof A, Tatari M, Christofori G. EMT as the ultimate survival mechanism of cancer cells. Semin Cancer Biol. 2012;22:194–207. 22406545 10.1016/j.semcancer.2012.02.013

[24] Glumac PM, LeBeau AM. The role of CD133 in cancer: a concise review. Clin Transl Med. 2018;7:18. 29984391 10.1186/s40169-018-0198-1 PMC6035906

[25] Atashzar MR, Baharlou R, Karami J, Abdollahi H, Rezaei R, Pourramezan F, et al. Cancer stem cells: a review from origin to therapeutic implications. J Cell Physiol. 2020;235:790–803. 31286518 10.1002/jcp.29044

[26] Zhou B, Lin W, Long Y, Yang Y, Zhang H, Wu K, et al. Notch signaling pathway: architecture, disease, and therapeutics. Signal Transduct Target Ther. 2022;7:95. 35332121 10.1038/s41392-022-00934-y PMC8948217

[27] Villanueva A, Alsinet C, Yanger K, Hoshida Y, Zong Y, Toffanin S, et al. Notch signaling is activated in human hepatocellular carcinoma and induces tumor formation in mice. Gastroenterology. 2012;143:1660–9.e7. 22974708 10.1053/j.gastro.2012.09.002 PMC3505826

[28] Shi Q, Xue C, Zeng Y, Yuan X, Chu Q, Jiang S, et al. Notch signaling pathway in cancer: from mechanistic insights to targeted therapies. Signal Transduct Target Ther. 2024;9:128. 38797752 10.1038/s41392-024-01828-x PMC11128457

[29] Sharan R, Maron-Katz A, Shamir R. CLICK and EXPANDER: a system for clustering and visualizing gene expression data. Bioinformatics. 2003;19:1787–99. 14512350 10.1093/bioinformatics/btg232

[30] Satija R, Farrell JA, Gennert D, Schier AF, Regev A. Spatial reconstruction of single-cell gene expression data. Nat Biotechnol. 2015;33:495–502. 25867923 10.1038/nbt.3192 PMC4430369

[31] Tickle T, Georgescu C, Tirosh I. Inferring CNV from Single-Cell RNA-Seq. GitHub. 2018. https://github.com/broadinstitute/inferCNV?tab=License-1-ov-file.

[32] Wickham H Data Analysis. In: Wickham H (ed). ggplot2: Elegant Graphics for Data Analysis. 2nd ed. Springer International Publishing: Cham, 2016.

[33] Kassambara A ggpubr: ‘ggplot2’ Based Publication Ready Plots (R Package Version 0.6.0). The Comprehensive R Archive Network. 2023. https://cran.r-project.org/web/packages/ggpubr/index.html.

