# Supplementary material for: Effect of Sortilin1 on promoting angiogenesis and systemic metastasis in hepatocellular carcinoma via the Notch signaling pathway and CD133
Source: Cell Death Dis. 2024 Aug 29;15(8):634. doi: 10.1038/s41419-024-07016-7 (PMC11362463; doi:10.1038/s41419-024-07016-7)
Supplement: Supplementary file 1 — Supplementary information [file 41419_2024_7016_MOESM1_ESM.docx]

**Supplementary Information**

**Supplementary** **Materials and Methods**

**Cell viability assessment**

Huh-7 and Hep3B cells were transfected with siRNA targeting *SORT1* for 24 h in 60 mm^2^ dishes. After transfection, the cells were trypsinized, stained with 0.4% trypan blue solution (Invitrogen), and counted using a hemocytometer (Paul Marienfeld GmbH & Co. KG, Lauda-Königshofen, Germany).

**Cell growth measurement using MTT Assay**

To evaluate cell growth, Huh-7 and Hep3B cells were seeded at a density of 1 × 10^4^ cells/well in 12-well plates and transfected with NC or siSORT1. After 24 h of incubation at 37°C, cells were treated with 50 μL 3‐(4,5‐dimethylthiazol‐2‐yl)‐2,5‐diphenyltetrazolium bromide (MTT) solution (1 mg/mL; Biosesang, Seongnam, South Korea) and incubated for 1 h at 37°C in the dark. Afterward, the supernatant was discarded, and formazan crystals were dissolved in 500 μL dimethyl sulfoxide (DMSO; Biosesang). The absorbance was recorded at 570 nm using a TECAN SUNRISE Microplate Reader (TECAN, Zürich, Switzerland).

**Clonogenic proliferation assay**

Following transfection with either NC or siSORT1, 1.5 × 10^5^ cells from each group were seeded onto 60 mm^2^ dishes for 24 h. The cells were then re-seeded into 6-well plates and cultivated at 37°C in a CO_2_ incubator for 15 d. After incubation, cells were fixed with 1% paraformaldehyde (Biosesang) for 30 min and stained with 0.5% crystal violet overnight at 22–25°C. Colony formation was quantified using ImageJ software (version 1.49; Laboratory for Optical and Computational Instrumentation, Madison, WI, USA).

**Cell cycle assay**

Huh-7 and Hep3B cells (1 × 10^5^) were seeded in 60 mm^2^ dishes and transfected with either a NC or siRNA targeting *SORT1*. After 24 h of incubation at 37°C, cells were harvested, fixed in 70% ethanol for 3 h at 4°C, and then centrifuged at 4,300 × g for 1 min at 4°C. The cell pellet was stained with a solution containing 10 μg/mL RNase A, 30 μg/mL propidium iodide (PI; Sigma-Aldrich), and 1% Triton X-100 (Sigma-Aldrich) in 200 μL v, followed by a 30-min incubation in the dark. The cell cycle distribution was assessed using a FACSAria III flow cytometer (BD Biosciences, San Jose, CA, USA).

**Apoptosis assay**

Huh-7 and Hep3B cells (1 × 10^5^) were plated in 60 mm^2^ dishes and transfected with NC or siSORT1 for 24 h. After transfection (48 h), the cells were trypsinized, rinsed with phosphate-buffered saline (PBS), and resuspended in 1× binding buffer. Cells were then labeled using the Annexin V-FITC Apoptosis Detection Kit (Koma Biotech, Seoul, South Korea) by combining with 1.25 μL Annexin V-FITC and 10 μL PI, kept in the dark and analyzed for apoptotic populations using a FACSAria III flow cytometer (BD Biosciences).

**Hoechst 33342/PI staining assay**

Cells were seeded at a density of 1 × 10^4^ cells/well in 12-well plates, transfected with NC or siSORT1, and incubated for 24 h. After transfection, cells were fixed with 4% paraformaldehyde for 10 min, followed by permeabilization with 0.2% Triton X-100 in 500 μL PBS for 20 min. After two washes with PBS, the cells were stained sequentially with Hoechst 33342 (10 μg/mL; Invitrogen) for 10 min, followed by PI (20 μg/mL) for 5 min. Changes in cell morphology were visualized via fluorescence microscopy using an Olympus IX71 microscope (Olympus, Tokyo, Japan).

**RNA Stability Assay**

To assess the stability of mRNA, Huh-7 and Hep3B cells were transfected with either siNC (negative control) or siSORT1 for 48 hours. Post-transfection, cells were treated with 10 μg/mL actinomycin D (Sigma-Aldrich) to inhibit transcription. Cells were harvested at 0, 1, 2, 4, 6 and 8 hours after actinomycin D treatment. Total RNA was extracted using the Qiazol reagent (Qiagen), and the levels of mRNA for *SORT1, NOTCH1, HPBJ, MAML1, MYC, HES1 and CCND3* and GAPDH were quantified by qRT-PCR. *GAPDH* was used as an internal control. RNA stability was calculated based on the decay rate of mRNA over time. The primers used for qRT-PCR are listed in Supplementary Table 5.

***Co-immunoprecipitation (co-IP)***

Transfected cells were washed with PBS and lysed at 4°C using lysis buffer composed of PBS (pH 7.2) containing 1.0% NP-40, 0.5% sodium deoxycholate, 0.1% SDS, 10 mmol/L NaF, 1.0 mmol/L NaVO_4_, and a 1.0% protease inhibitor cocktail (Sigma-Aldrich), following a previously described protocol (Ebiomedicine 2016:9;97-109). Equal protein aliquots (1.0 mg) were subjected to immunoprecipitation with 2.0 μg of specific antibodies against CD133 (Abcam, ab19898), using protein A/G-agarose (Santa Cruz Biotechnology). The immunoprecipitated proteins were separated on 10% SDS-polyacrylamide gels and transferred to polyvinylidene fluoride membranes (Bio-Rad Laboratories). After blocking with PBS containing 0.1% Tween 20 (PBS-T) and 5% non-fat dry milk (Sigma-Aldrich) for 1 h, the membranes were incubated with antibodies against anti-CD133 (Abcam, ab19898), anti-ADAM10 (Abcam, ab124695), and anti-ADAM17 (Proteintech, IL, USA, 29948-1-AP) at a dilution of 1:1,000. Following washing with PBS-T, the membranes were incubated with horseradish peroxidase-conjugated anti-mouse (Sigma-Aldrich) or anti-rabbit immunoglobulin G (IgG) antibody (Sigma-Aldrich) at a dilution of 1:5,000 for 1 h at room temperature. Protein bands were visualized using a luminol-based enhanced chemiluminescence plus Western blotting detection system (Amersham Biosciences, Buckinghamshire, UK). The identification of immunoreactive bands was achieved by co-migration with pre-stained protein size markers (Fermentas, Glen Burnie, MD, USA).

**5-aza-2'-deoxycytidine Treatment**

HCC cells were treated with the methylation inhibitor 5-aza-2'-deoxycytidine (5-aza, Sigma-Aldrich). The cells were cultured in the presence of 5 μM 5-aza for 48 hours at 37°C in a CO_2_ incubator, with the culture medium replaced daily. After treatment, the cells were harvested for the detection of *SORT1* methylation and expression levels. *SORT1* mRNA expression was measured using qRT-PCR, protein levels were evaluated by Western blot analysis, and methylation levels were analyzed using quantitative methylation-specific PCR (qMSP).

**Supplementary Tables**

**Supplementary Table** **1**: Homogeneity results for each cluster in the three datasets assessed using the CLICK algorithm

| GSE6764 | | | |
| --- | --- | --- | --- |
| No | Cluster | Gene | Homogeneity |
| 1 | Cluster_1 | 182 | 0.751 |
| 2 | Cluster_2 | 133 | 0.701 |
| 3 | Cluster_3 | 45 | 0.456 |
| 4 | Cluster_4 | 31 | 0.685 |
|  |  |  |  |
| GSE54238 | | | |
| No | Cluster | Gene | Homogeneity |
| 1 | Cluster_1 | 243 | 0.738 |
| 2 | Cluster_2 | 135 | 0.722 |
|  |  |  |  |
| GSE589377 | | | |
| No | Cluster | Gene | Homogeneity |
| 1 | Cluster_1 | 242 | 0.664 |
| 2 | Cluster_2 | 135 | 0.687 |

**Supplementary Table 2:** Differential expression of common 58 driver genes in five HCC RNA-seq datasets

|  | TCGA | | |  | ICGC | | |  | GSE77314 | | |  | GSE114564 | | |  | GSE124535 | | | |
| --- | --- | --- | --- | --- | --- | --- | --- | --- | --- | --- | --- | --- | --- | --- | --- | --- | --- | --- | --- | --- |
| GENE | *p* value |  | Fold change (log2) |  | *p* value |  | Fold change (log2) |  | *p* value |  | Fold change (log2) |  | *p* value |  | Fold change (log2) |  | *p* value |  | Fold change (log2) |  |
| *ZWINT* | 6.6E-50 |  | 1.912 |  | 3.0E-32 |  | 6.032 |  | 5.3E-34 |  | 2.536 |  | 2.8E-11 |  | 1.149 |  | 6.1E-11 |  | 1.945 |  |
| *YWHAZ* | 6.7E-26 |  | 1.075 |  | 1.9E-24 |  | 2.202 |  | 2.0E-11 |  | 0.970 |  | 2.5E-10 |  | 0.676 |  | 6.1E-11 |  | 1.320 |  |
| *VPS45* | 2.0E-55 |  | 1.208 |  | 5.7E-57 |  | 2.317 |  | 3.8E-10 |  | 0.690 |  | 3.5E-20 |  | 0.811 |  | 1.8E-12 |  | 0.982 |  |
| *UBE2T* | 2.5E-73 |  | 2.326 |  | 1.4E-43 |  | 8.949 |  | 1.6E-29 |  | 2.663 |  | 3.8E-11 |  | 1.254 |  | 1.6E-12 |  | 2.050 |  |
| *UBE2C* | 1.4E-83 |  | 2.610 |  | 5.3E-24 |  | 11.129 |  | 3.1E-30 |  | 3.795 |  | 6.8E-09 |  | 1.272 |  | 2.7E-10 |  | 2.537 |  |
| *TYMS* | 1.2E-32 |  | 1.595 |  | 2.9E-27 |  | 2.752 |  | 5.0E-17 |  | 1.576 |  | 7.2E-09 |  | 1.115 |  | 1.8E-08 |  | 1.588 |  |
| *TPX2* | 2.5E-50 |  | 1.735 |  | 2.1E-31 |  | 4.555 |  | 1.6E-26 |  | 2.357 |  | 3.1E-12 |  | 1.258 |  | 1.4E-08 |  | 1.568 |  |
| *TK1* | 2.2E-46 |  | 2.232 |  | 6.4E-24 |  | 4.884 |  | 4.0E-22 |  | 2.148 |  | 1.2E-05 |  | 0.779 |  | 3.1E-08 |  | 1.487 |  |
| *TGM3* | 2.5E-62 |  | 1.826 |  | 1.6E-18 |  | 34.345 |  | 5.3E-07 |  | 1.501 |  | 1.5E-07 |  | 0.918 |  | 4.3E-04 |  | 1.186 |  |
| *TARBP1* | 6.6E-73 |  | 1.397 |  | 2.1E-49 |  | 3.063 |  | 3.9E-10 |  | 1.116 |  | 3.4E-20 |  | 1.252 |  | 5.7E-12 |  | 1.431 |  |
| *SULT1C2* | 1.9E-48 |  | 1.089 |  | 1.9E-14 |  | 11.861 |  | 9.6E-13 |  | 2.566 |  | 1.1E-06 |  | 1.223 |  | 1.0E-07 |  | 2.084 |  |
| *STMN1* | 1.2E-54 |  | 1.972 |  | 3.1E-43 |  | 4.575 |  | 7.9E-25 |  | 2.495 |  | 7.9E-11 |  | 1.293 |  | 6.1E-13 |  | 2.007 |  |
| *STIP1* | 3.5E-50 |  | 1.370 |  | 7.5E-35 |  | 2.182 |  | 1.2E-19 |  | 1.425 |  | 1.7E-13 |  | 0.696 |  | 9.2E-10 |  | 1.084 |  |
| *SQLE* | 2.0E-19 |  | 1.998 |  | 2.3E-21 |  | 3.621 |  | 7.5E-17 |  | 2.476 |  | 6.4E-09 |  | 1.396 |  | 1.6E-06 |  | 1.740 |  |
| *SPINK1* | 9.3E-11 |  | 2.662 |  | 1.0E-10 |  | 9.938 |  | 4.8E-10 |  | 4.208 |  | 1.0E-07 |  | 2.721 |  | 1.0E-07 |  | 4.829 |  |
| *SORT1* | 4.1E-17 |  | 1.366 |  | 2.7E-29 |  | 3.486 |  | 1.0E-10 |  | 1.113 |  | 1.5E-04 |  | 0.638 |  | 1.6E-08 |  | 1.335 |  |
| *SMC4* | 4.4E-40 |  | 0.841 |  | 2.1E-31 |  | 2.663 |  | 5.6E-14 |  | 1.170 |  | 4.1E-13 |  | 0.979 |  | 2.1E-09 |  | 1.403 |  |
| *SETDB1* | 6.9E-53 |  | 0.968 |  | 8.2E-49 |  | 2.292 |  | 7.2E-12 |  | 0.746 |  | 2.5E-16 |  | 0.685 |  | 1.0E-10 |  | 0.772 |  |
| *RFX5* | 4.8E-27 |  | 1.363 |  | 6.8E-44 |  | 2.759 |  | 1.2E-08 |  | 0.812 |  | 2.9E-10 |  | 0.706 |  | 3.4E-14 |  | 1.670 |  |
| *RFC4* | 6.0E-71 |  | 1.476 |  | 3.1E-37 |  | 3.564 |  | 9.5E-27 |  | 1.892 |  | 5.8E-11 |  | 1.001 |  | 7.0E-11 |  | 1.576 |  |
| *RACGAP1* | 7.5E-84 |  | 1.481 |  | 6.5E-42 |  | 5.933 |  | 1.3E-22 |  | 1.837 |  | 9.4E-14 |  | 1.275 |  | 4.2E-11 |  | 1.687 |  |
| *PTTG1* | 8.1E-95 |  | 2.302 |  | 1.0E-27 |  | 7.830 |  | 1.2E-33 |  | 3.500 |  | 2.2E-11 |  | 1.552 |  | 1.2E-11 |  | 2.203 |  |
| *PRIM1* | 4.6E-45 |  | 1.089 |  | 7.4E-35 |  | 3.113 |  | 3.1E-14 |  | 1.123 |  | 5.9E-10 |  | 0.788 |  | 2.1E-07 |  | 1.085 |  |
| *PRC1* | 2.5E-95 |  | 1.604 |  | 2.6E-40 |  | 8.307 |  | 2.7E-32 |  | 2.740 |  | 7.7E-12 |  | 1.486 |  | 5.1E-10 |  | 1.820 |  |
| *PODXL* | 6.2E-35 |  | 1.393 |  | 8.1E-23 |  | 2.987 |  | 2.5E-06 |  | 0.713 |  | 3.4E-09 |  | 0.753 |  | 1.2E-11 |  | 1.628 |  |
| *PEG10* | 1.8E-23 |  | 1.779 |  | 9.5E-09 |  | 20.254 |  | 1.1E-11 |  | 3.582 |  | 1.9E-05 |  | 1.697 |  | 1.4E-04 |  | 2.451 |  |
| *NUSAP1* | 6.8E-61 |  | 1.838 |  | 4.1E-43 |  | 6.062 |  | 2.0E-24 |  | 2.198 |  | 5.4E-12 |  | 1.475 |  | 5.2E-11 |  | 2.026 |  |
| *NSMCE2* | 5.5E-66 |  | 1.449 |  | 2.7E-44 |  | 3.077 |  | 2.1E-20 |  | 1.438 |  | 2.4E-19 |  | 0.849 |  | 3.0E-15 |  | 1.381 |  |
| *NQO1* | 1.6E-33 |  | 2.562 |  | 1.2E-09 |  | 19.817 |  | 8.7E-08 |  | 2.704 |  | 1.6E-04 |  | 1.298 |  | 4.3E-05 |  | 1.933 |  |
| *NCAPD2* | 1.6E-43 |  | 1.258 |  | 5.0E-30 |  | 3.046 |  | 4.6E-14 |  | 0.860 |  | 3.7E-08 |  | 0.652 |  | 6.3E-07 |  | 0.963 |  |
| *MCM7* | 8.6E-56 |  | 1.662 |  | 1.2E-34 |  | 2.803 |  | 5.7E-21 |  | 1.368 |  | 1.5E-07 |  | 0.631 |  | 3.3E-10 |  | 1.237 |  |
| *MCM4* | 4.2E-52 |  | 1.504 |  | 7.0E-32 |  | 3.968 |  | 3.6E-15 |  | 1.715 |  | 1.8E-10 |  | 0.853 |  | 6.4E-10 |  | 1.800 |  |
| *MCM3* | 2.4E-52 |  | 1.760 |  | 1.0E-36 |  | 3.379 |  | 3.9E-26 |  | 2.009 |  | 7.4E-10 |  | 0.784 |  | 2.5E-13 |  | 1.485 |  |
| *LAPTM4B* | 7.2E-36 |  | 1.587 |  | 1.1E-21 |  | 4.140 |  | 1.1E-12 |  | 2.116 |  | 8.5E-04 |  | 0.660 |  | 5.6E-07 |  | 1.757 |  |
| *LAMC1* | 1.2E-47 |  | 1.571 |  | 7.9E-24 |  | 3.663 |  | 2.3E-11 |  | 1.575 |  | 5.9E-11 |  | 0.797 |  | 4.9E-10 |  | 1.384 |  |
| *KNTC1* | 1.8E-59 |  | 0.748 |  | 3.6E-24 |  | 2.996 |  | 5.2E-12 |  | 1.403 |  | 6.5E-14 |  | 1.189 |  | 5.2E-09 |  | 1.142 |  |
| *KIF2C* | 2.1E-89 |  | 1.470 |  | 1.4E-31 |  | 8.626 |  | 3.3E-25 |  | 2.361 |  | 7.8E-10 |  | 1.030 |  | 2.0E-07 |  | 1.310 |  |
| *KIF20A* | 3.5E-91 |  | 1.454 |  | 6.7E-36 |  | 9.258 |  | 4.9E-29 |  | 2.418 |  | 6.2E-12 |  | 1.141 |  | 3.9E-09 |  | 1.365 |  |
| *IGSF3* | 2.0E-48 |  | 1.035 |  | 7.2E-24 |  | 7.082 |  | 1.0E-13 |  | 1.116 |  | 2.6E-08 |  | 0.893 |  | 5.9E-09 |  | 1.333 |  |
| *IGF2BP2* | 1.8E-32 |  | 1.141 |  | 4.3E-14 |  | 3.725 |  | 1.8E-09 |  | 1.468 |  | 5.1E-08 |  | 1.103 |  | 6.1E-06 |  | 1.513 |  |
| *GPC3* | 1.9E-57 |  | 4.739 |  | 1.4E-19 |  | 22.009 |  | 3.9E-20 |  | 4.779 |  | 4.9E-14 |  | 3.286 |  | 8.3E-09 |  | 4.384 |  |
| *G6PD* | 3.9E-61 |  | 1.832 |  | 1.6E-09 |  | 5.066 |  | 3.9E-13 |  | 2.227 |  | 2.0E-04 |  | 0.600 |  | 3.0E-06 |  | 1.202 |  |
| *FOXM1* | 1.1E-86 |  | 1.631 |  | 1.2E-32 |  | 4.478 |  | 2.1E-20 |  | 1.740 |  | 1.8E-13 |  | 1.334 |  | 4.5E-08 |  | 1.496 |  |
| *FEN1* | 9.0E-64 |  | 1.719 |  | 6.4E-39 |  | 3.690 |  | 3.8E-23 |  | 1.959 |  | 1.9E-08 |  | 0.788 |  | 2.8E-11 |  | 1.391 |  |
| *FANCG* | 4.2E-63 |  | 1.115 |  | 4.1E-36 |  | 2.806 |  | 4.1E-11 |  | 1.043 |  | 4.7E-08 |  | 0.626 |  | 1.7E-05 |  | 0.812 |  |
| *FAM83D* | 1.2E-61 |  | 1.681 |  | 2.0E-25 |  | 6.648 |  | 4.4E-20 |  | 1.771 |  | 2.5E-10 |  | 1.061 |  | 7.1E-10 |  | 1.420 |  |
| *EZH2* | 1.4E-83 |  | 1.340 |  | 1.0E-37 |  | 3.622 |  | 1.7E-26 |  | 1.845 |  | 1.3E-13 |  | 1.019 |  | 3.2E-11 |  | 1.497 |  |
| *EXO1* | 2.3E-82 |  | 0.928 |  | 5.1E-36 |  | 9.126 |  | 2.2E-21 |  | 1.847 |  | 4.2E-12 |  | 1.093 |  | 1.9E-08 |  | 1.166 |  |
| *ESM1* | 6.5E-87 |  | 1.708 |  | 1.9E-25 |  | 15.239 |  | 6.3E-20 |  | 1.714 |  | 6.9E-10 |  | 1.193 |  | 5.5E-11 |  | 1.685 |  |
| *COL4A1* | 2.8E-29 |  | 1.949 |  | 1.6E-17 |  | 2.922 |  | 3.2E-09 |  | 1.400 |  | 1.7E-04 |  | 0.699 |  | 1.6E-12 |  | 2.169 |  |
| *CDC25C* | 1.6E-93 |  | 1.119 |  | 1.9E-37 |  | 8.822 |  | 5.0E-23 |  | 1.705 |  | 2.1E-09 |  | 0.965 |  | 1.0E-08 |  | 1.048 |  |
| *CDC20* | 5.0E-96 |  | 2.418 |  | 3.7E-24 |  | 11.437 |  | 2.2E-31 |  | 3.770 |  | 3.8E-09 |  | 1.147 |  | 6.5E-08 |  | 1.685 |  |
| *CCNB2* | 3.0E-98 |  | 1.710 |  | 2.2E-31 |  | 8.940 |  | 5.5E-27 |  | 2.740 |  | 7.3E-11 |  | 1.218 |  | 1.3E-09 |  | 1.831 |  |
| *CCNA2* | 3.1E-76 |  | 1.715 |  | 2.8E-29 |  | 3.773 |  | 6.6E-21 |  | 1.434 |  | 2.6E-11 |  | 1.093 |  | 4.1E-10 |  | 1.403 |  |
| *AURKA* | 6.3E-70 |  | 1.942 |  | 1.9E-32 |  | 8.297 |  | 1.4E-23 |  | 2.226 |  | 9.7E-10 |  | 1.204 |  | 1.4E-10 |  | 1.702 |  |
| *AKR1C3* | 5.4E-42 |  | 1.918 |  | 2.2E-34 |  | 4.116 |  | 3.4E-06 |  | 1.206 |  | 2.5E-13 |  | 1.299 |  | 4.9E-07 |  | 1.242 |  |
| *ACSL4* | 2.8E-17 |  | 2.316 |  | 1.7E-22 |  | 9.168 |  | 4.2E-15 |  | 2.578 |  | 1.0E-07 |  | 1.591 |  | 7.7E-10 |  | 3.229 |  |
| *ABCC4* | 3.1E-31 |  | 0.738 |  | 5.1E-19 |  | 3.318 |  | 8.3E-07 |  | 0.662 |  | 1.6E-10 |  | 1.017 |  | 2.7E-08 |  | 1.263 |  |

TCGA, the cancer genome atlas; ICGC, the international cancer genome consortium

**Supplementary Table 3**: Clinicopathological characteristics of the patients in the Ajou Hospital (AJOU-HCC) cohort

| Characteristics | | *n* = 86 |
| --- | --- | --- |
| Age, years | | 56.28 |
| Gender (male) | | 68 (79.07) |
| Etiology | |  |
|  | HBV | 80 (93.02) |
|  | HCV | 5 (5.81) |
|  | Alcohol | 1 (1.16) |
| Cirrhosis | | 44 (51.16) |
| Platelet, x 10^3^/μL | | 174.06 (64.67) |
| Albumin, g/dL | | 4.47 (0.69) |
| Total bilirubin, mg/dL | | 0.85 (1.06) |
| Creatinine, mg/dL | | 0.87 (0.23) |
| AST, IU/L | | 50.80 (79.60) |
| ALT, IU/L | | 45.77 (55.64) |
| AFP (>200 ng/mL) | | 22 (25.58) |
| PIVKA-II (>40 mAU/mL) | | 56 (65.12) |
| Macrovascular invasion | | 25 (29.07) |
| Lymph node metastasis | | 2 (2.33) |
| Distant metastasis | | 3 (3.49) |
| mUICC stage | |  |
|  | I | 24 (27.91) |
|  | II | 34 (39.53) |
|  | III | 22 (25.58) |
|  | IV | 1 (1.16) |
|  | IVA | 2 (2.33) |
|  | IVB | 3 (3.49) |

Data were expressed as number (%). AST, aspartate aminotransferase; ALT, alanine transaminase; AFP, alpha-fetoprotein; PIVKA-II, protein induced by vitamin k absense or antagonist-II; UICC, Union for International Cancer Control

**Supplementary Table 4**: Primary and secondary antibodies used in the study

|  | **Antibody** | **Species** | **Dilution** | **Manufacturer** | **Catalog No.** |
| --- | --- | --- | --- | --- | --- |
| **IHC** | |  |  |  |  |
|  | SORT1 | Rabbit | 1:100 | Abcam | ab16640 |
|  | Ki-67 | Mouse | 1:500 | Abcam | ab15580 |
|  | PCNA | Mouse | 1:10,000 | Cell Signaling Technology | 2586 |
|  | CD31 | Rabbit | 1:500 | Abcam | ab134168 |
|  | VEGF | Mouse | 1:100 | Santa Cruz Biotechnology | sc-7269 |
|  | ZO-1 | Mouse | 1:200 | Thermo Fisher Scientific | 33-9100 |
|  | Vimentin | Rabbit | 1:500 | GeneTex | GTX100619 |
|  | **Antibody** | **Species** | **Dilution** | **Manufacturer** | **Catalog No.** |
| **Western blot** | | | | | |
|  | SORT1 | Rabbit | 1:1,000 | Abcam | ab16640 |
|  | p-Wee1 | Rabbit | 1:1,000 | Cell Signaling Technology | 4910 |
|  | Cyclin B1 | Mouse | 1:500 | Santa Cruz Biotechnology | sc-245 |
|  | Cyclin D1 | Rabbit | 1:1,000 | Cell Signaling Technology | 9932 |
|  | Cyclin D3 | Mouse | 1:2,000 | Cell Signaling Technology | 9932 |
|  | Cdc2 | Rabbit | 1:1,000 | Cell Signaling Technology | 9112 |
|  | Cdc25A | Rabbit | 1:1,000 | Cell Signaling Technology | 3652 |
|  | p53 | Mouse | 1:1,000 | Santa Cruz Biotechnology | sc-126 |
|  | PARP | Rabbit | 1:1,000 | Cell Signaling Technology | 9915 |
|  | Cleaved PARP | Rabbit | 1:1,000 | Cell Signaling Technology | 9915 |
|  | Caspase-3 | Rabbit | 1:1,000 | Cell Signaling Technology | 9915 |
|  | Cleaved Caspase-3 | Rabbit | 1:1,000 | Cell Signaling Technology | 9915 |
|  | Caspase-9 | Mouse | 1:1,000 | Cell Signaling Technology | 9915 |
|  | Cleaved Caspase-9 | Rabbit | 1:1,000 | Cell Signaling Technology | 9915 |
|  | ZO-1 | Mouse | 1:1,000 | Thermo Fisher Scientific | 33-9100 |
|  | E-cadherin | Mouse | 1:1,000 | BD bioscience | 610404 |
|  | Fibronectin | Mouse | 1:1,000 | Santa Cruz Biotechnology | sc-8422 |
|  | N-cadherin | Mouse | 1:2,000 | BD bioscience | 610920 |
|  | Vimentin | Rabbit | 1:5,000 | GeneTex | GTX100619 |
|  | Snail | Rabbit | 1:1,000 | Cell Signaling Technology | 3879S |
|  | CD133 | Rabbit | 1:1,000 | Abcam | ab19898 |
|  | Notch1 | Rabbit | 1:500 | Proteintech | 20687-1-AP |
|  | Cleaved Notch1 | Rabbit | 1:1,000 | Cell Signaling Technology | 68309 |
|  | RBPSUH | Rabbit | 1:1,000 | Cell Signaling Technology | 68309 |
|  | MAML1 | Rabbit | 1:1,000 | Cell Signaling Technology | 68309 |
|  | c-Myc | Rabbit | 1:1,000 | Cell Signaling Technology | 68309 |
|  | HES1 | Rabbit | 1:1,000 | Cell Signaling Technology | 68309 |
|  | GAPDH | Mouse | 1:1,000 | Santa Cruz Biotechnology | sc-32233 |
|  | **Secondary antibody** | | **Dilution** | **Manufacturer** | **Catalog No.** |
|  | Goat anti-Rabbit IgG H&L (HRP) | | 1:3,000 | Bio-Rad Laboratories | 170-6515 |
|  | Goat anti-Mouse IgG H&L (HRP) | | 1:3,000 | Bio-Rad Laboratories | 170-6516 |

**Supplementary Table 5**: Primary and secondary antibodies used in the study

| No | Gene | Primer | Primer seqeance | Tm (℃) |
| --- | --- | --- | --- | --- |
| 1 | *NOTH1* | F | TGGACCAGATTGGGGAGTTC | 56 |
|  |  | R | GCACACTCGTCTGTGTTGAC |  |
| 2 | *HPBJ* | F | CAGCGCCTTCAACAGGTTTC | 58 |
|  |  | R | GGGTTTTAGGACGCGCTTTG |  |
| 3 | *MAML1* | F | CTCCGTCTCAGCTGCCTATG | 56 |
|  |  | R | TGCTGCCAGGAGGAATTCTG |  |
| 4 | *MYC* | F | ACCACCAGCAGCGACTCTGA | 56 |
|  |  | R | TCCAGCAGAAGGTGATCCAGACT |  |
| 5 | *HES1* | F | GTCAACACGACACCGGATAA | 56 |
|  |  | R | TTCAGCTGGCTCAGACTTTC |  |
| 6 | *CCND3* | F | AGAGCACCAGCCCCTTTGTC | 62 |
|  |  | R | ATCCGAACAGAGCCAGTCTCC |  |

**Supplementary Figure**


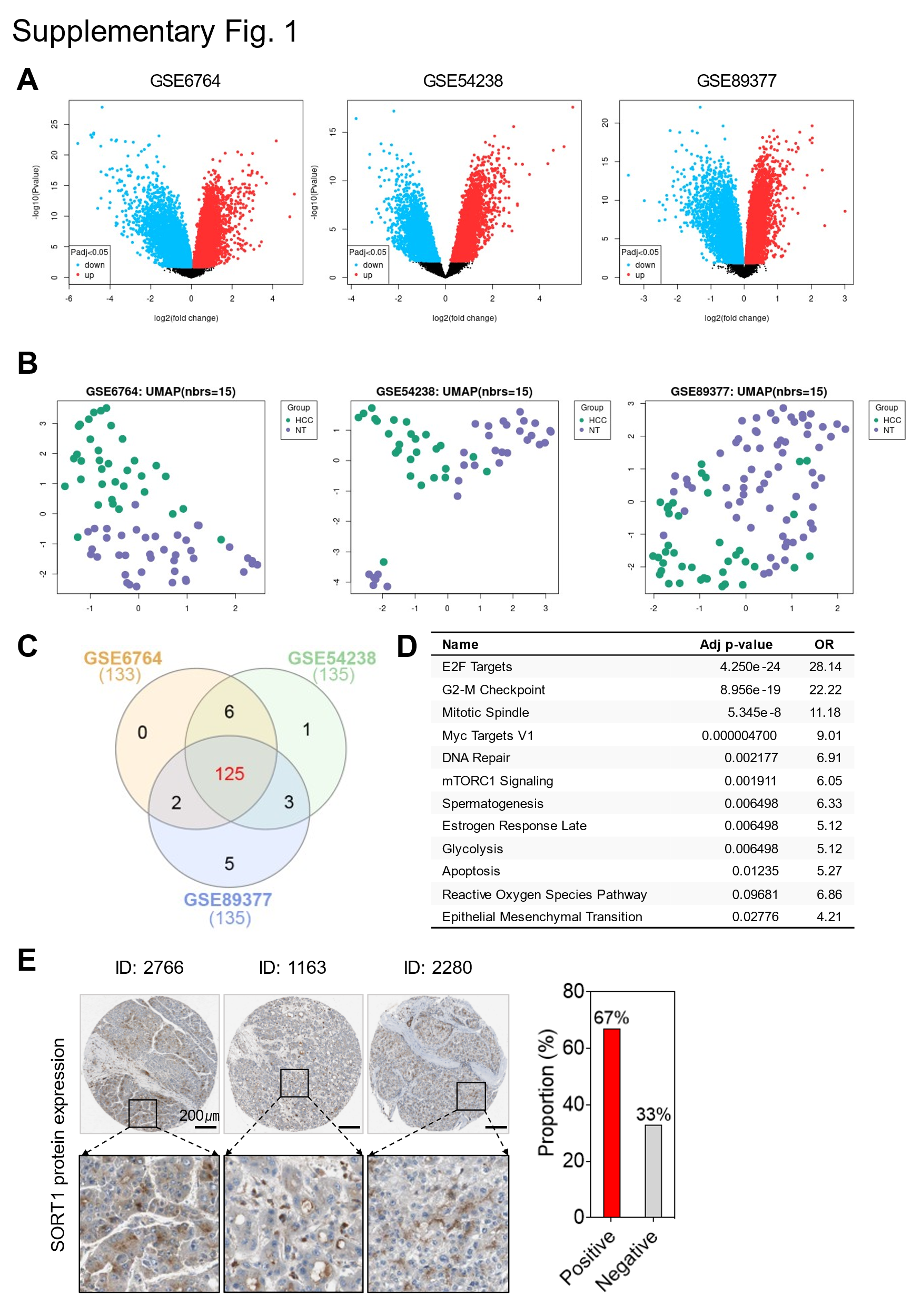


**Supplementary Fig. 1: Extended analysis and validation of the identified** **hepatocellular carcinoma (HCC) driver gene signatures and their clinical significance.**

**A** Volcano plots for each dataset (GSE6764, GSE54238, and GSE89377) displaying significantly differentially expressed genes. These plots highlight genes of interest based on statistical significance and magnitude of expression change. **B** Uniform Manifold Approximation and Projection (UMAP) plots illustrating the distribution of HCC and NT samples across three different datasets: GSE6764, GSE54238, and GSE89377. Each plot showcases the distinct clustering of HCC and non-tumoral (NT) samples. **C** Venn diagram representing the overlap of genes identified in the three datasets (GSE6764, GSE54238, and GSE89377). **D** Table detailing the pathways associated with the identified genes. The table lists pathways, their respective adjusted p-values, and odds ratios (OR). The most significant pathways include E2F Targets, G2–M Checkpoint, and Mitotic Spindle, among others.

Statistical significance in the graph is indicated by ***, representing *p* < 0.001. The analysis was performed using a Student's *t*-test. **E** Representative immunohistochemistry (IHC) images and bar chart showing SORT1 staining ratios in human HCC tissues and adjacent non-tumor tissues. IHC images highlight the significant overexpression of SORT1 in malignant HCC tissues compared to non-tumorous tissues, with a higher staining intensity and frequency.

**
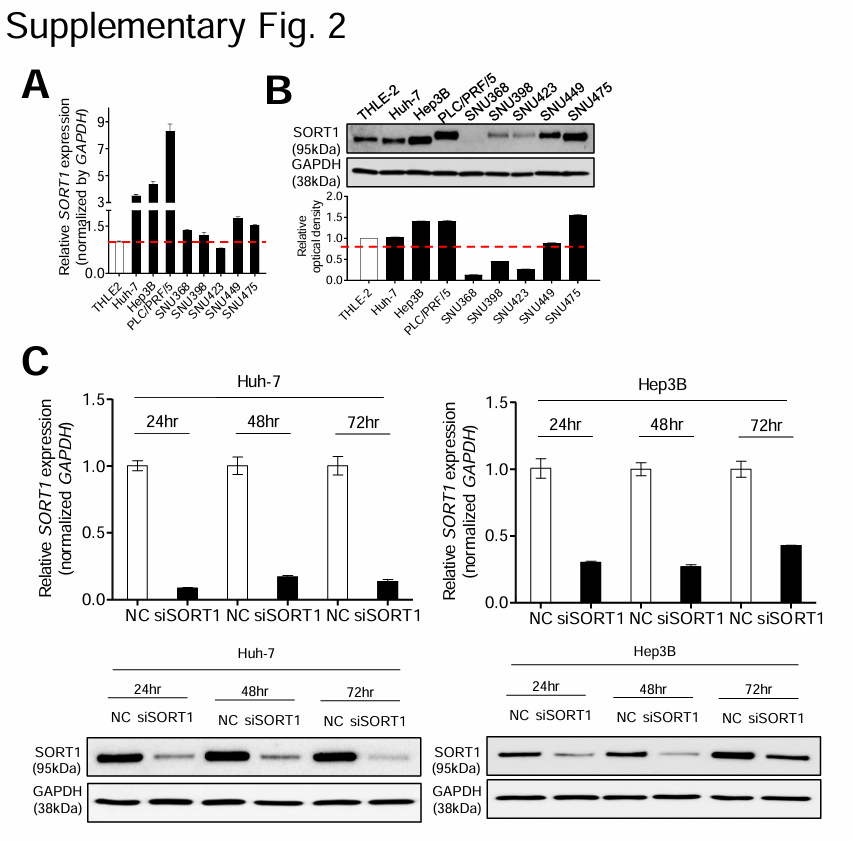
**

**Supplementary Fig. 2: Evaluation of SORT1 gene and protein expression in hepatocellular carcinoma (HCC) cell lines.**

**A-B** Quantitative real-time polymerase chain reaction (qRT-PCR) **(A)** and western blot analyses **(B)** of SORT1 expression across various liver cell lines. The bar graph represents mRNA levels, and the blots display the corresponding protein expression levels. GAPDH serves as a control. **C** qRT-PCR and western blot analysis of SORT1 expression in Huh-7 and Hep3B liver cancer cell lines after silencing of SORT1 at various time points (24, 48, and 72 h). Protein bands represent the levels of SORT1 and GAPDH (as a loading control).

Statistical significance is indicated as ** *p* < 0.01, *** *p* < 0.001. The analysis was performed using a Student's t-test and Welch’s *t*-test.


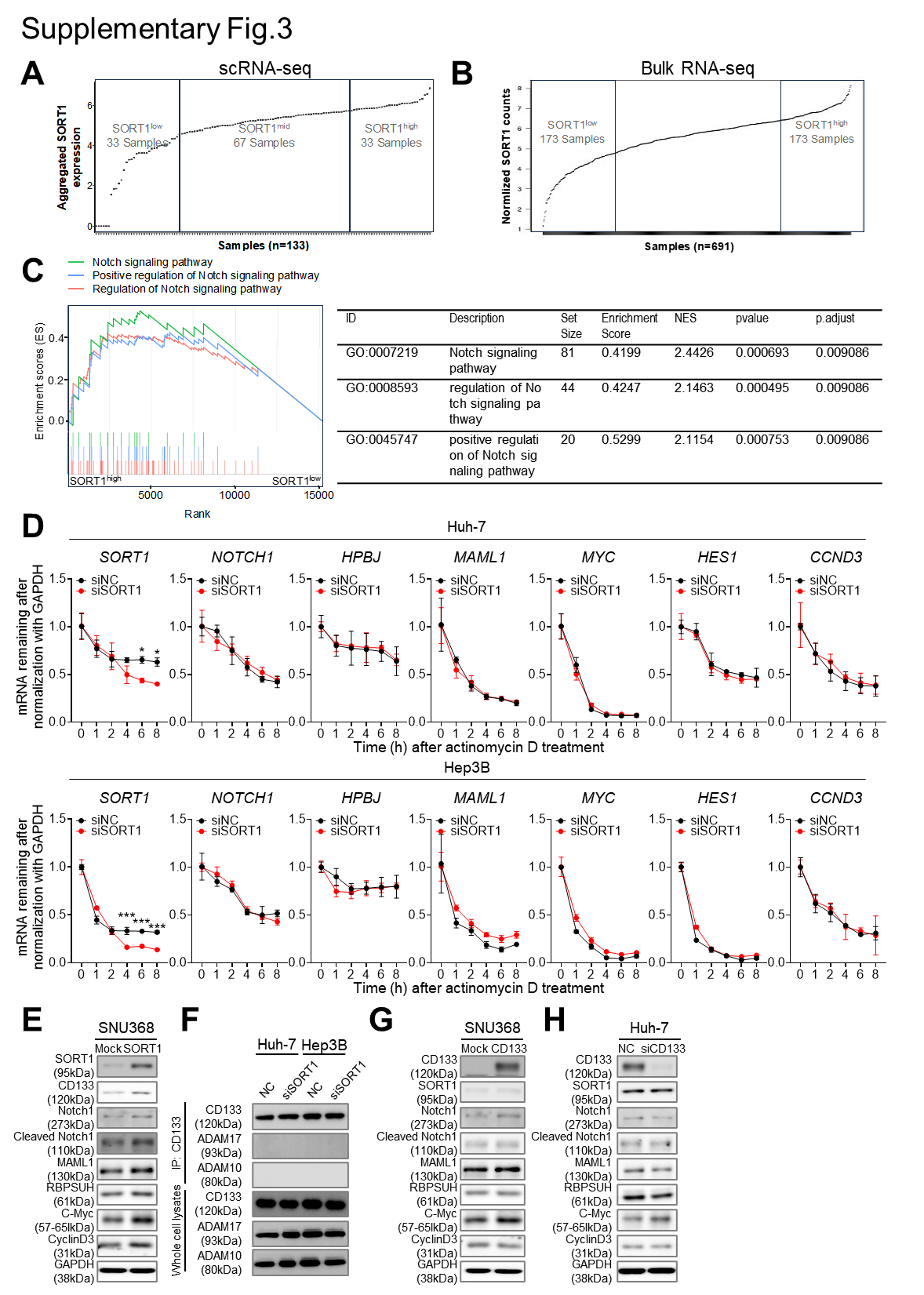


**Supplementary Fig. 3: Analysis of *SORT1* Expression and Notch Signaling Pathway**

**A** Pseudo-bulk analysis of scRNA-seq data, showing aggregated *SORT1* expression stratified into *SORT1*^low^, *SORT1*^mid^, and *SORT1*^high^ groups (n=133). **B** Bulk RNA-seq data analysis with samples stratified into *SORT1*^low^, *SORT1*^mid^, and *SORT1*^high^ groups (n=173). **C** Gene Set Enrichment Analysis of Notch signaling pathway gene sets ranked by normalized enrichment score (NES). **D** mRNA stability assessment of *NOTCH1*, *HES1*, *MAML1*, *MYC* and *CCDN3* in Huh-7 and Hep3B cells transfected with siNC or siSORT1, treated with Actinomycin D (10 μg/mL) at indicated time points. RNA levels were measured using qRT-PCR and normalized to *GAPDH*. **E** Overexpression of SORT1 in Huh-7 and Hep3B cells resulted in increased cleavage of Notch1 and activation of downstream Notch signaling components. **F** Co-immunoprecipitation results showing no direct binding interaction between CD133 and ADAM10 or ADAM17. **G** Rescue experiment showing that overexpression of CD133 in SORT1-deficient SNU368 cells did not result in significant changes in SORT1 or dramatic cleavage of Notch1, nor did it activate the downstream Notch pathway. **H** Knockdown experiment in Huh-7 cells, demonstrating that reducing CD133 levels did not affect SORT1 expression or downstream Notch signaling.

**
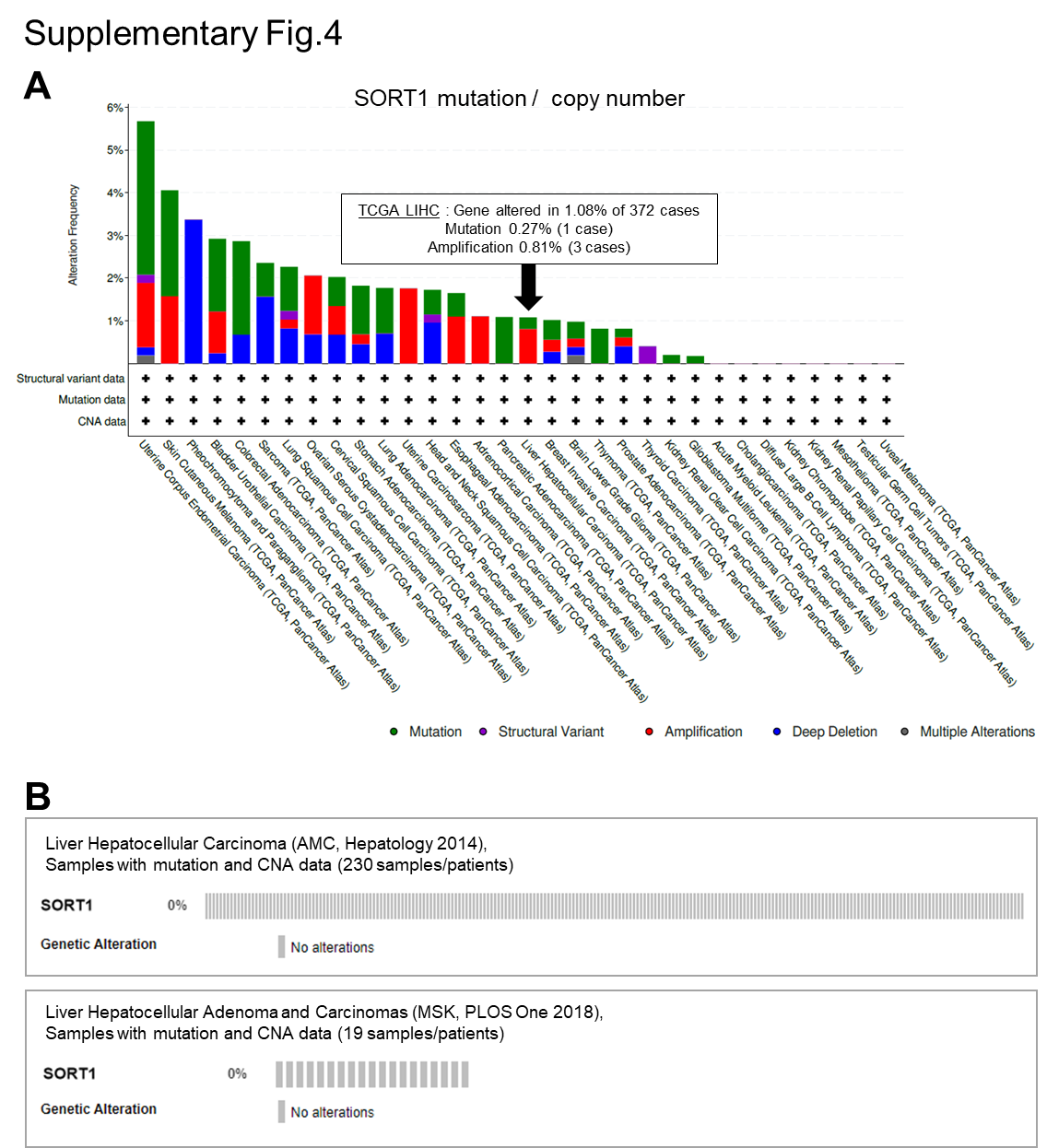
**

**Supplementary Fig. 4: Analysis of *SORT1* mutations and copy number alterations in liver cancers.**

**A** Distribution of *SORT1* mutation and copy number alterations in The Cancer Genome Atlas Liver Hepatocellular Carcinoma (TCGA LIHC) dataset. The graph represents the frequency of structural variants, mutations, and copy number alterations (CNA) data, detailing specific alterations. The dataset indicated that *SORT1* was altered in 1.08% of 372 cases, with a mutation in 0.27% (1 case) and amplification in 0.81% (3 cases). **B** Genetic alteration analysis of *SORT1* in liver cancers, utilizing data from two specific studies available on cBioPortal: 'Liver Hepatocellular Carcinoma' from Asan Medical Center (AMC), published in Hepatology in 2014, and 'Liver Hepatocellular Adenoma and Carcinomas' from Memorial Sloan Kettering (MSK), published in PLOS One in 2018.

Statistical significance is indicated as * *p* < 0.05; ** *p* < 0.01. The analysis was performed using a Welch’s *t*-test and one-way ANOVA.


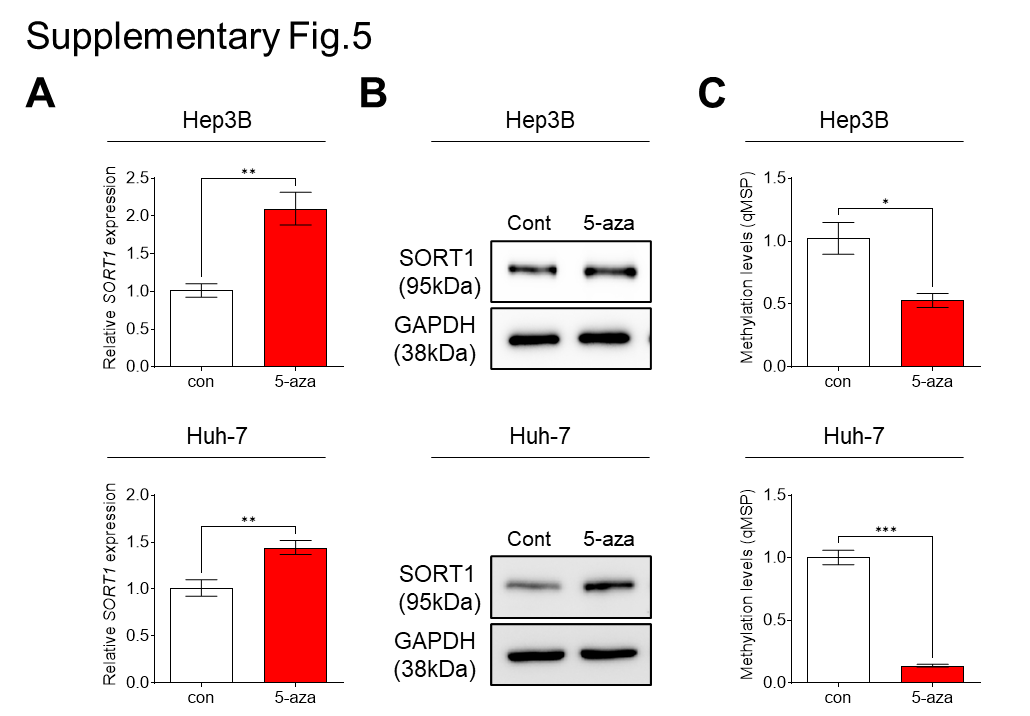


Supplementary Fig. 5: Effects of 5-aza treatment on SORT1 expression and methylation levels in HCC cells. **A** Relative expression levels of *SORT1* in control and 5-aza treated cells, assessed by qRT-PCR and normalized to *GAPDH*. **B** Western blot analysis showing the protein expression levels of SORT1 in control and 5-aza treated cells, with GAPDH as a loading control. **C** Methylation levels of *SORT1* in control and 5-aza treated cells, assessed by qMSP. Data are presented as mean ± SD. Statistical significance is indicated as * *p* < 0.05; ** *p* < 0.01; *** *p* < 0.001. The analysis was performed using a Welch’s *t*-test.
