# Supplementary figures and images for: Effect of Sortilin1 on promoting angiogenesis and systemic metastasis in hepatocellular carcinoma via the Notch signaling pathway and CD133
Source: Cell Death Dis. 2024 Aug 29;15(8):634. doi: 10.1038/s41419-024-07016-7 (PMC11362463; doi:10.1038/s41419-024-07016-7)

**Full and uncropped western blots**


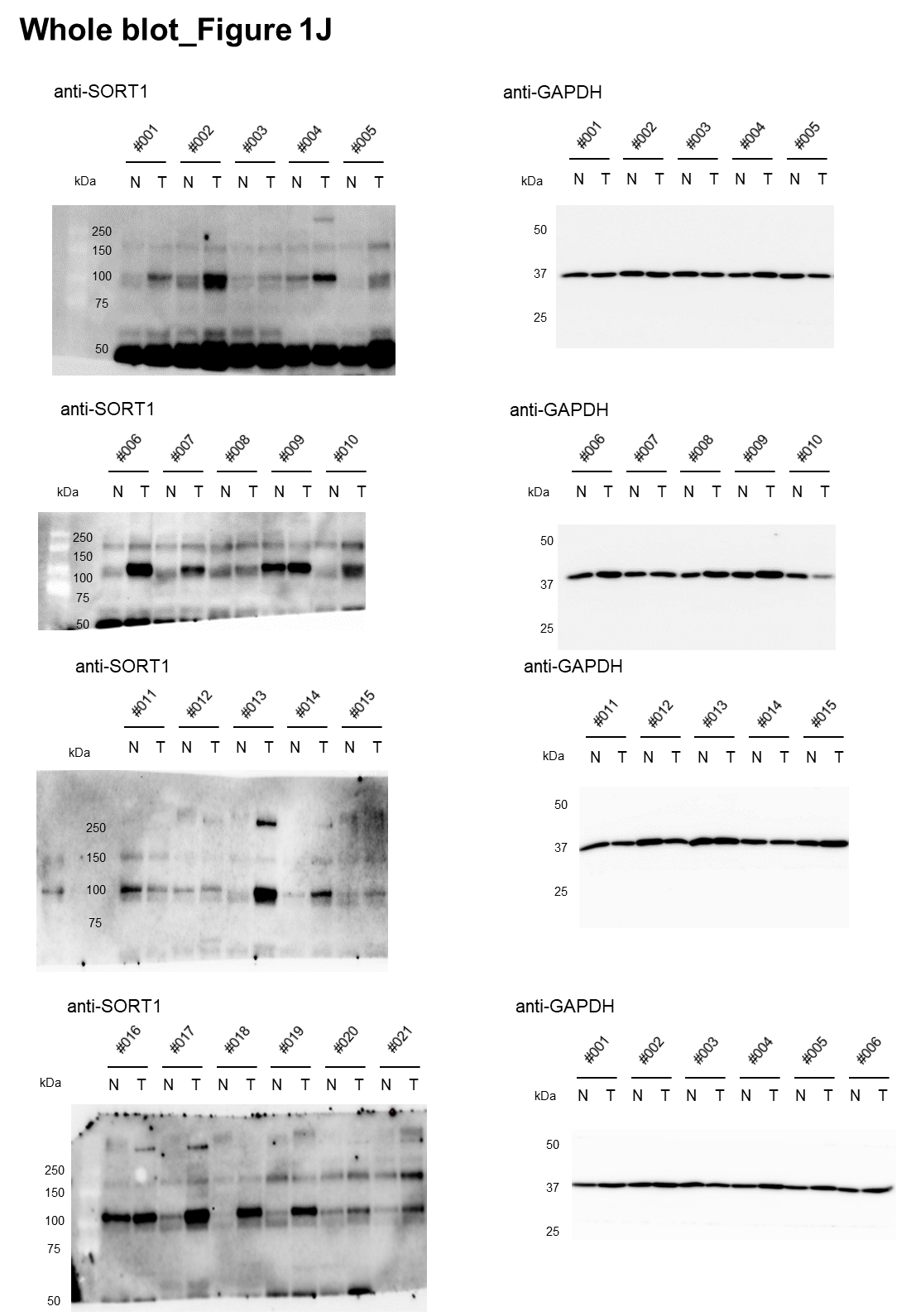


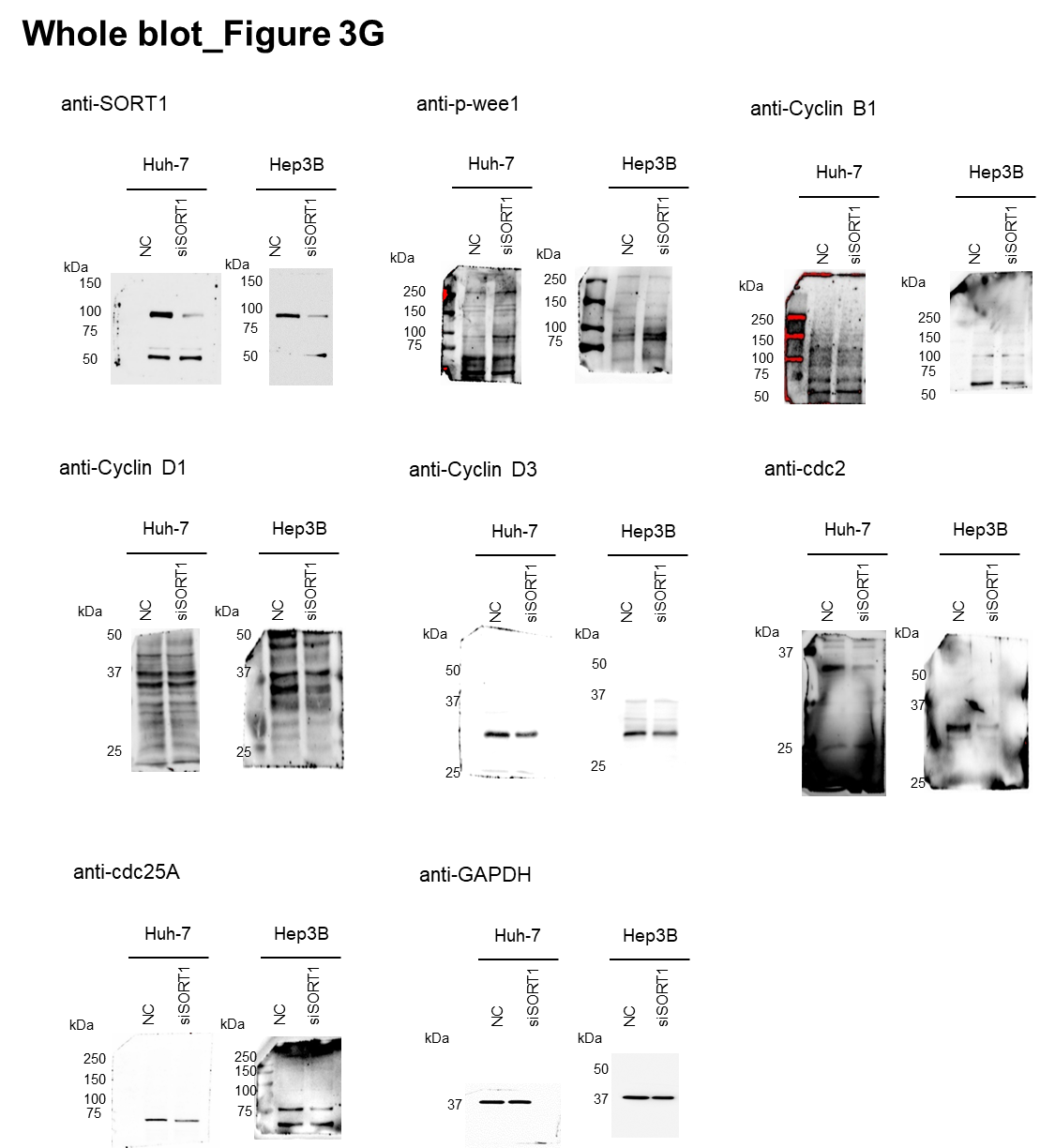


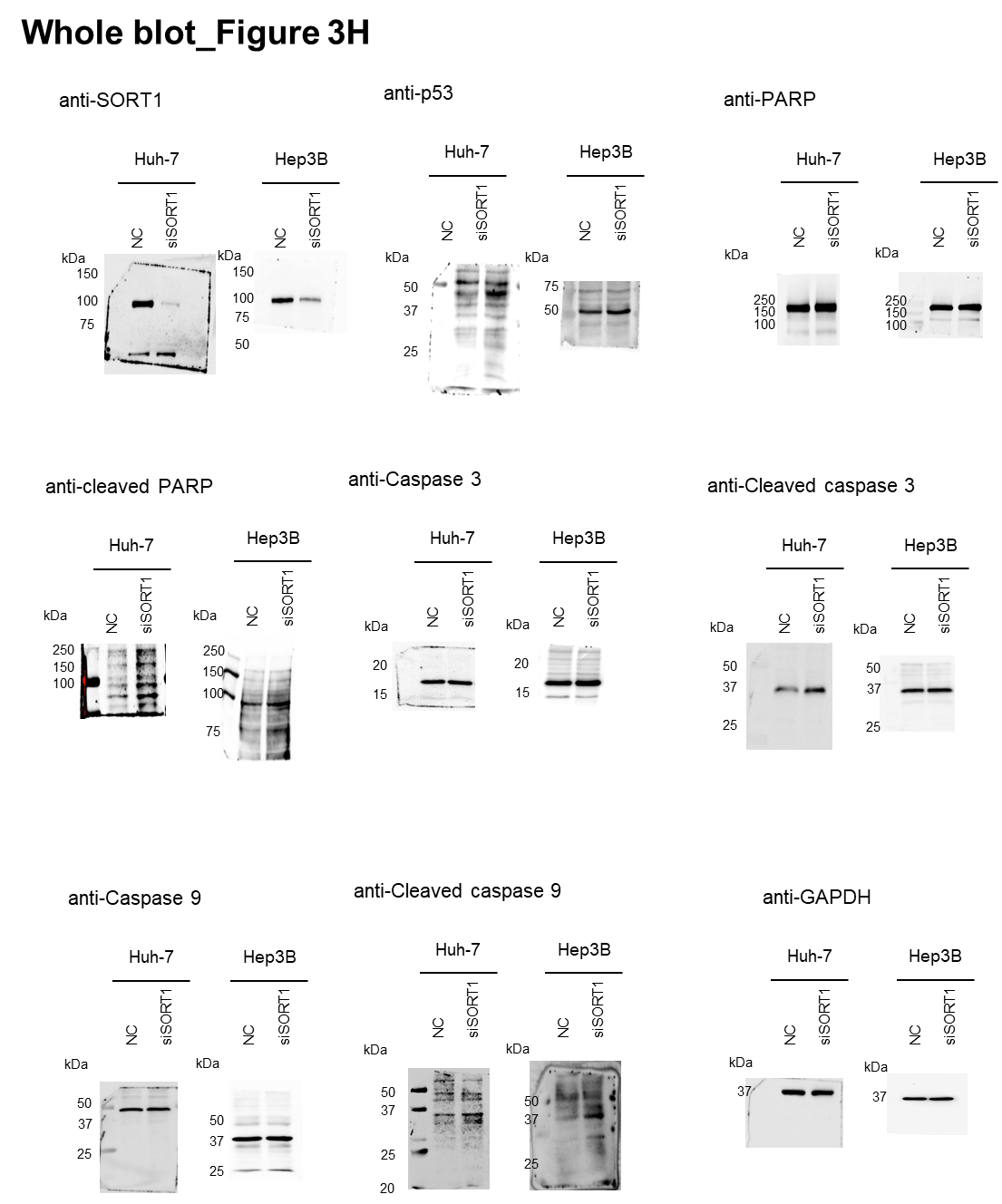


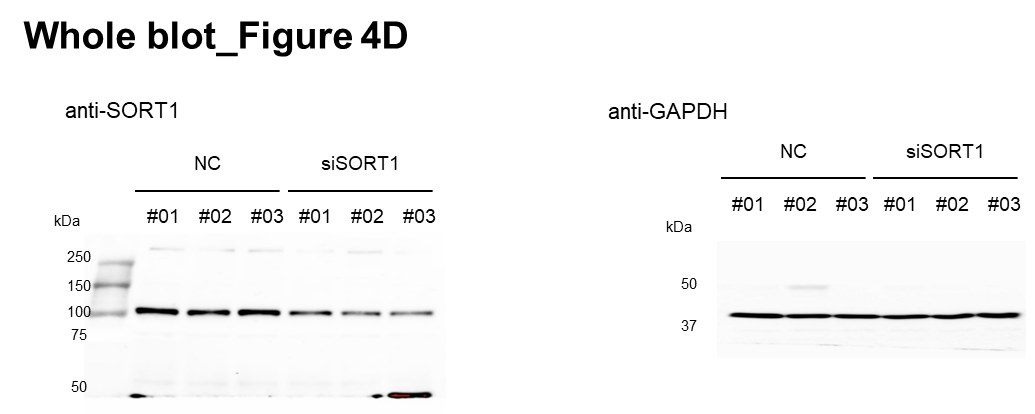


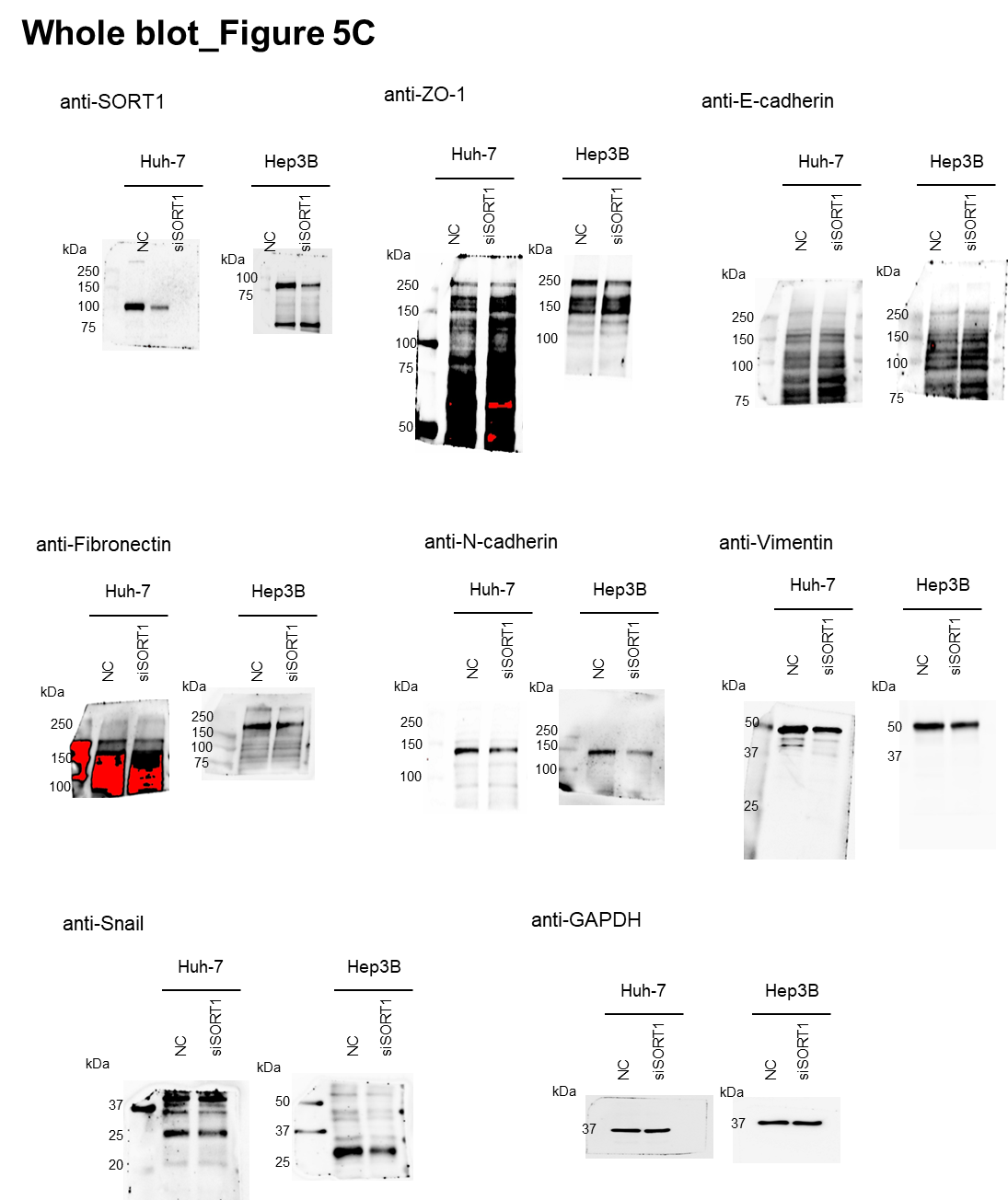


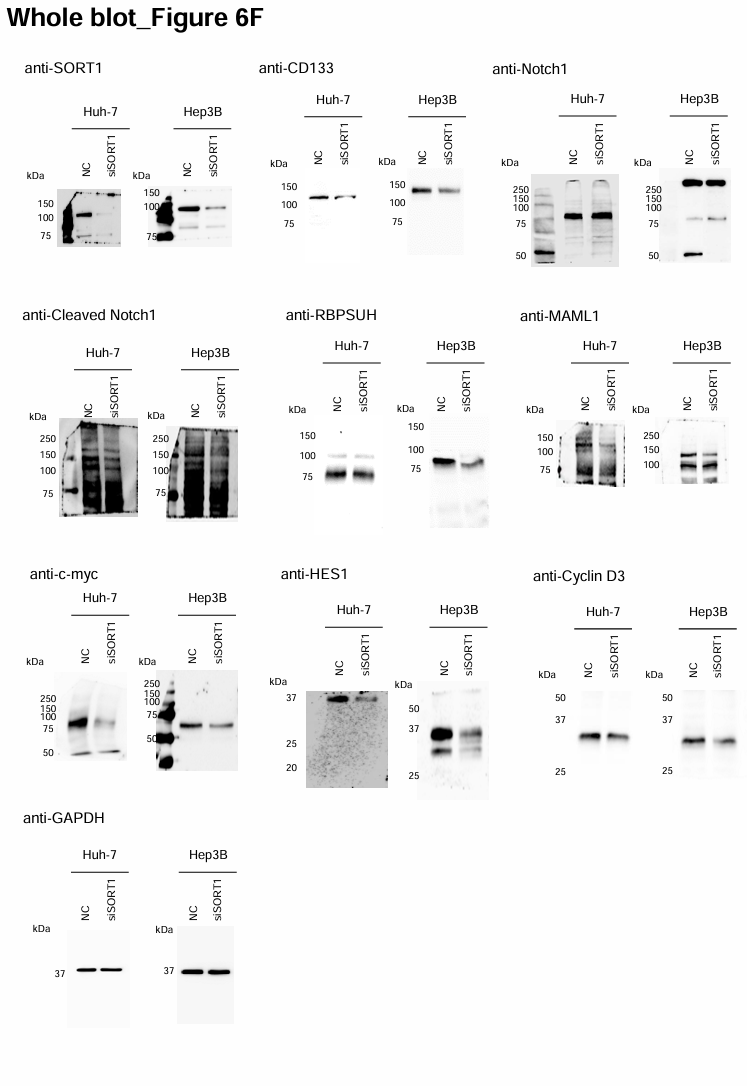


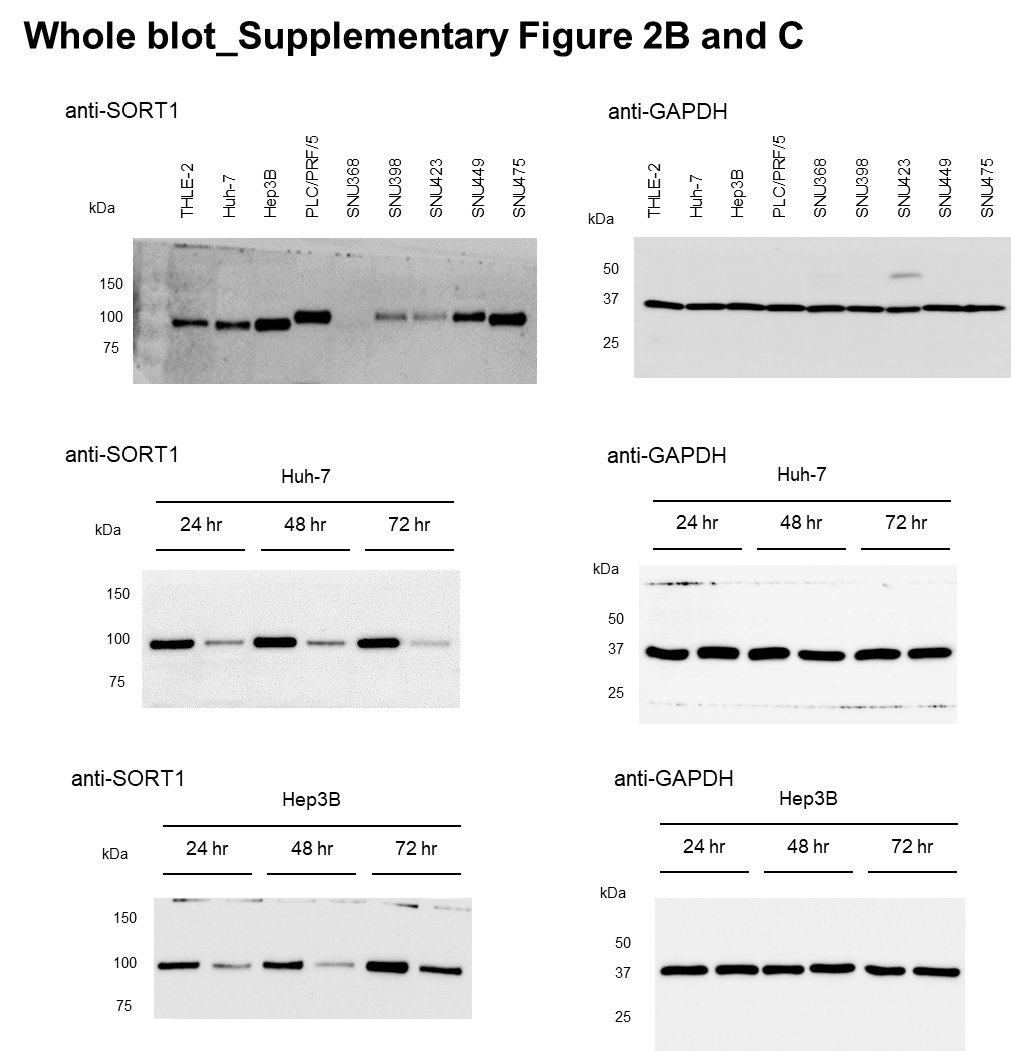


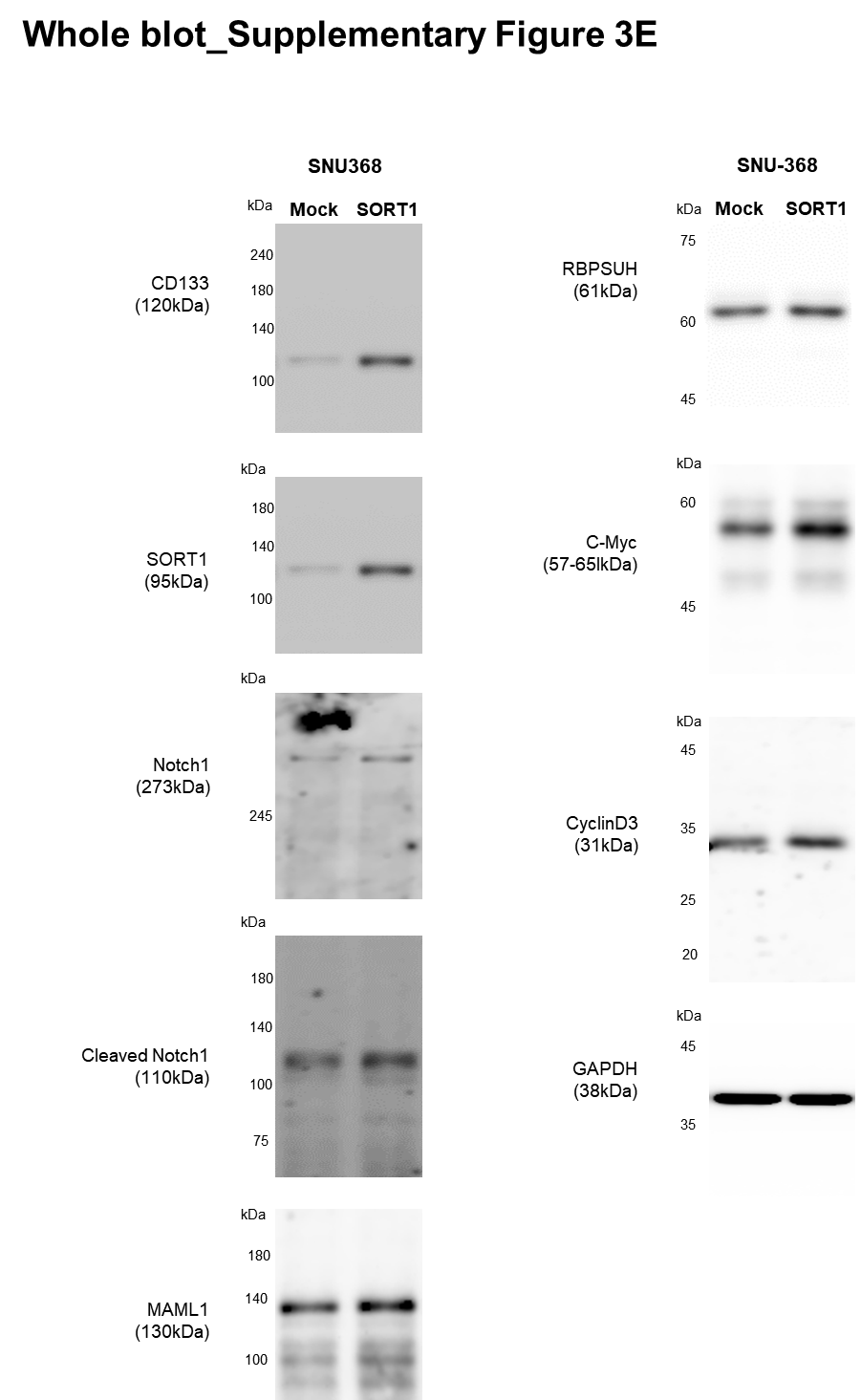


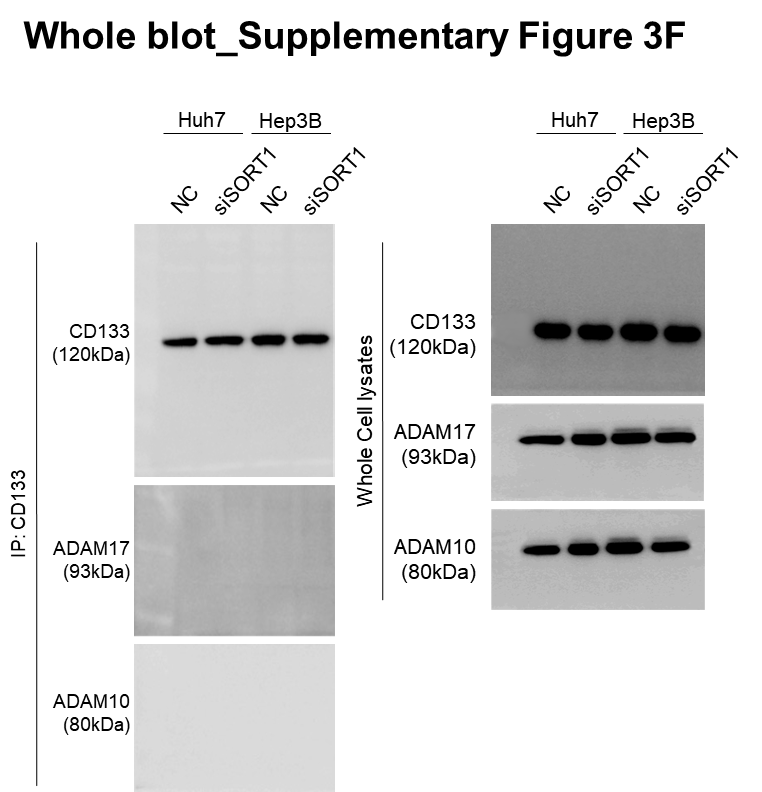


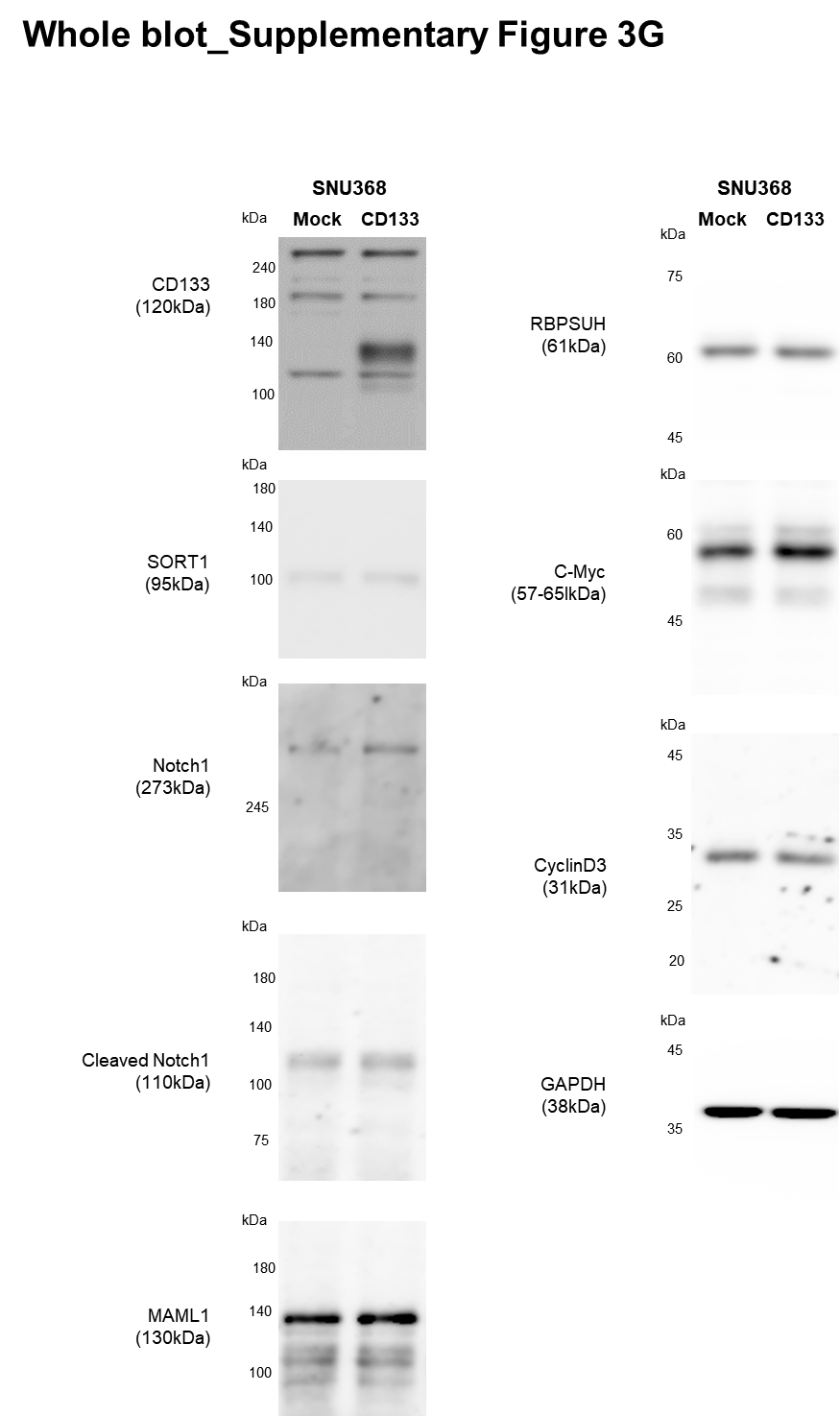


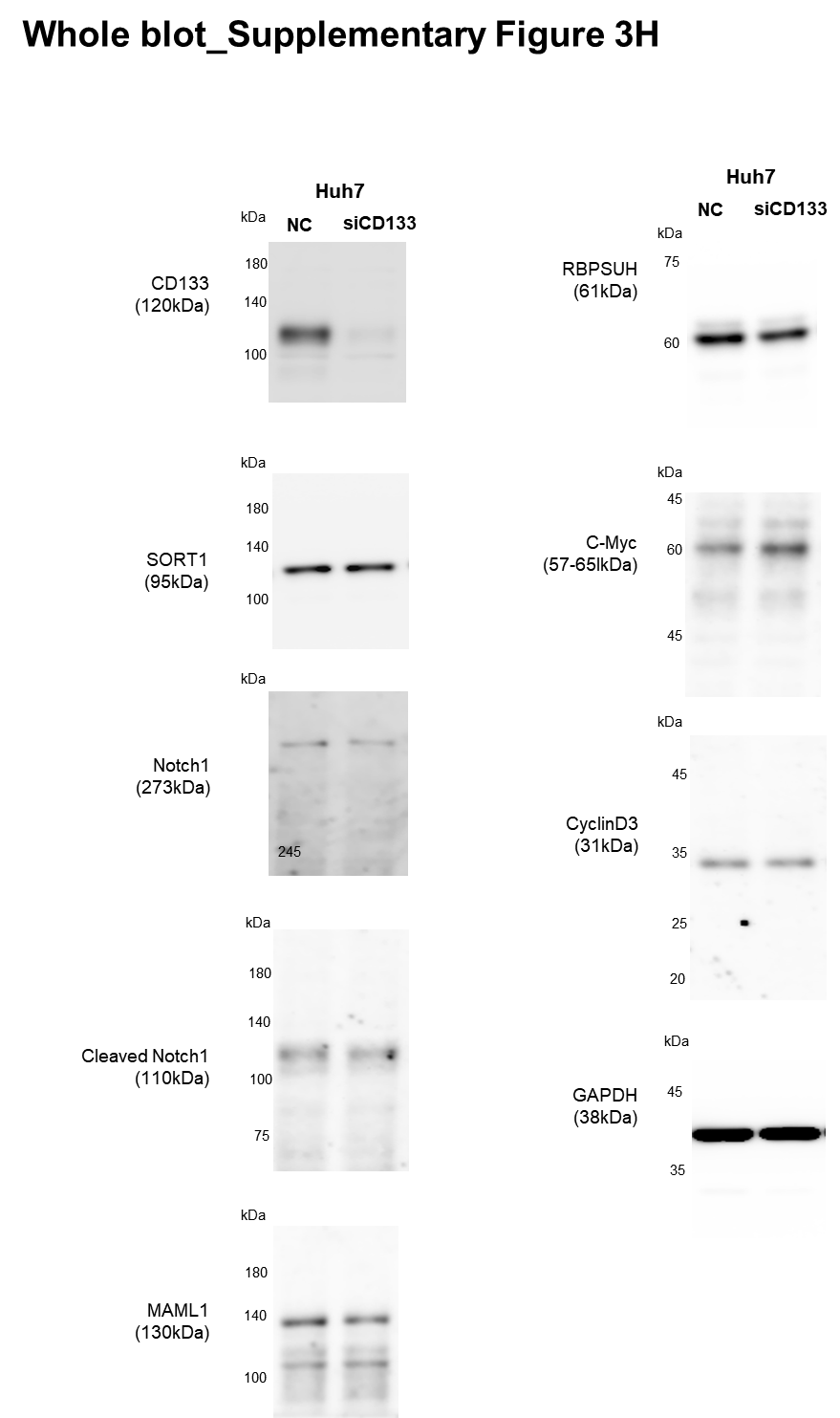


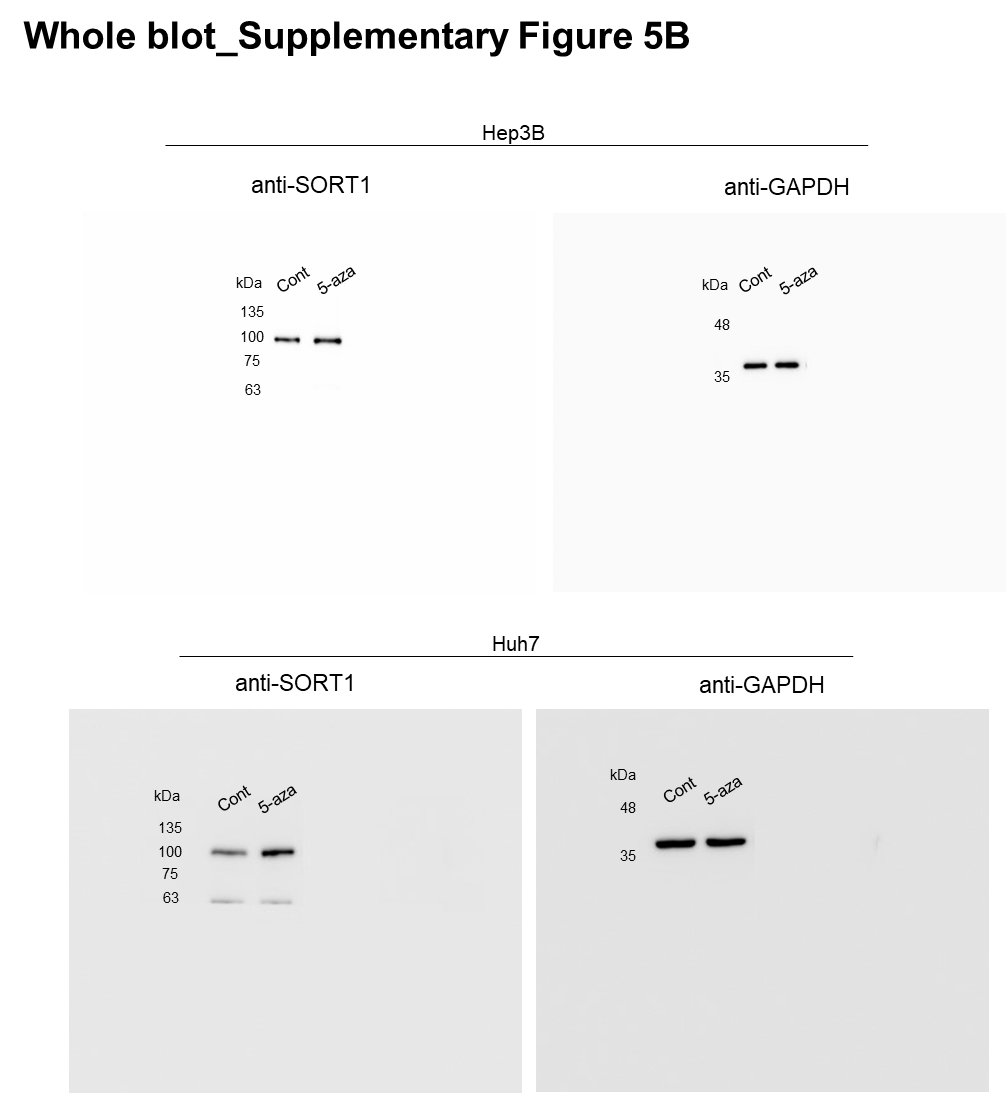

Supplement: Supplementary file 2 — Original Data [file 41419_2024_7016_MOESM2_ESM.docx]
